# Multivariate Shortfall and Divergence Risk Statistics

**DOI:** 10.3390/e21111031

**Published:** 2019-10-24

**Authors:** Haiyan Song, Xianfu Zeng, Yanhong Chen, Yijun Hu

**Affiliations:** 1 School of Mathematics and Statistics, Wuhan University, Wuhan 430072, China; xfzeng@whu.edu.cn (X.Z.); yjhu.math@whu.edu.cn (Y.H.); 2 College of Finance and Statistics, Hunan University, Changsha 410082, China; yhchen@hnu.edu.cn

**Keywords:** risk measures, entropic risk measures, convex risk statistics, (multivariate) shortfall risk statistics, (multivariate) divergence risk statistics, entropy-like risk statistics, representation results, 91B30, 91B32, 91B70

## Abstract

The aim of this paper is to construct two new classes of multivariate risk statistics, and to study their properties. We, first, introduce the multivariate shortfall risk statistics and multivariate divergence risk statistics. Then, their basic properties are studied, and their representation results are provided. Furthermore, their coherency is also characterized by means of the corresponding loss function. Finally, entropic risk statistics are given to illustrate the proposed new classes of multivariate risk statistics. The relationship between multivariate shortfall and divergence risk statistics is also discussed.

## 1. Introduction

To evaluate the risk of financial positions, in paper [1], the authors first introduced the concept of coherent risk measure. In [2,3], the authors introduced the broader class, named convex risk measure.

Shortfall risk measures and divergence risk measures are two important kinds of risk measures, and they have a dual relationship, as pointed out by [4] and [5]. Shortfall risk measures were introduced by [2] for random variables. For more studies about shortfall risk measures, see [4,5,6,7,8,9] and the references therein. Divergence risk measures were introduced by [10]. For more works about divergence risk measures, see [5,8,11] and the references therein.

For a financial portfolio $\tilde{X}=\left(X_{1},\cdots ,X_{N}\right)$ consisting of *N* financial positions, to measure not only the risk of the marginals $X_{i}$ separately, but also the joint risk of all components $X_{i}$ caused by their possible dependence between $X_{i}$. In [12], the authors first introduced the scalar multivariate coherent and convex risk measures, see also [13]. For more works on multivariate risk measures, see [8,9,14,15,16,17] and the references therein.

From the statistical point of view, the behaviour of a random variable can be characterized by its observations: the samples of the random variable. In [18,19], the authors first introduced the natural risk statistic, which can be considered as a data-based (or empirical) version of a coherent risk measure. In [20,21], the authors studied convex and quasiconvex risk statistics, respectively. In [22,23], the authors studied multivariate convex risk statistics.

In the aforementioned frameworks of risk statistics, the approaches are mainly axiomatic ones, and mainly focus on the representation results for various kinds of risk statistics. An natural and interesting issue is how about constructing meaningful risk statistics. Taking into account the importance of the shortfall risk measures, in the present paper, we attempt to explore the multivariate shortfall risk statistics, as well as the multivariate divergence risk statistics. This consideration mainly motivates the present study.

The purpose of the present paper is to construct multivariate shortfall and divergence risk statistics, respectively, and to study their properties, including their representation results. We provide the representation results for the multivariate shortfall and divergence risk statistics with explicit penalty functions, which are expressed in terms of the corresponding loss functions or divergence functions, respectively. The coherency of the univariate shortfall risk statistics is also characterized by means of the corresponding loss function. Finally, as examples, the multivariate entropic (or entropy-like) risk statistics are constructed to illustrate the proposed multivariate shortfall and divergence risk statistics.

The steps and methods of the present paper are as follows. We, first, introduce the acceptance set of the accepted portfolios. Then, the multivariate shortfall risk statistics are introduced, and their properties including their representations are investigated. Further, the coherency of the univariate shortfall risk statistics is characterized. Meanwhile, a kind of multivariate risk statistic closely related the multivariate shortfall risk statistic is also introduced and investigated, which is the so-called multivariate divergence risk statistic. Finally, examples are given. Convex analysis is employed to complete the involved argumentation.

The main contribution of the present paper are as follows. First, we have constructed two new meaningful classes of multivariate risk statistics, which are multivariate shortfall and divergence risk statistics. Second, their properties including the representation results are investigated, and their coherency is characterized. Finally, multivariate entropic (or entropy-like) risk statistics are introduced.

The rest of the paper is organized as follows. In Section 2, we briefly state some preliminaries including the definitions of multivariate shortfall and divergence risk measures. The main results are stated in Section 3. In Section 4, All the proofs of the main results of the paper are provided. In Section 5, examples are given. Finally, conclusions are summarized.

## 2. Preliminaries

In this section, we briefly introduce some preliminaries. Henceforth, let N≥1 be a fixed positive integer. We describe the loss of a financial position by a random variable. In practice, the behavior of a random loss vector $\tilde{X}=\left(X_{1},\cdots ,X_{N}\right)$ under different scenarios is preferably represented by different sets of data generated or observed under those scenarios because specifying accurate models for $\tilde{X}$ (under different scenarios) is usually very difficult. Assume that there exist *K* scenarios. Let $n_{ij}$ be the sample size of $X_{i}$ in the *j*th scenario for any 1≤i≤N and 1≤j≤K. Denote $n_{i}:=\sum_{j=1}^{K}n_{ij}$ for any 1≤i≤N and $n:=\sum_{i=1}^{N}n_{i}$. That is, the behavior of $\tilde{X}=\left(X_{1},\cdots ,X_{N}\right)$ is represented by a collection of data $\tilde{x}=\left({\tilde{x}}_{1},\cdots ,{\tilde{x}}_{N}\right)\in R^{n_{1}}\times \cdots \times R^{n_{N}}=R^{n}$, where ${\tilde{x}}_{i}=\left({\tilde{x}}^{i,1},\cdots ,{\tilde{x}}^{i,K}\right)\in R^{n_{i1}}\times \cdots \times R^{n_{iK}}=R^{n_{i}}$ and ${\tilde{x}}^{i,j}=\left(x_{1}^{i,j},\cdots ,x_{n_{ij}}^{i,j}\right)\in R^{n_{ij}}$ is the data subset that corresponds to the *j*th scenario of $X_{i}$. For each i=1,⋯,N, j=1,⋯,K, ${\tilde{x}}^{i,j}$ can be a data set based on historical observations of $X_{i}$, hypothetical samples simulated according to a model, or a mixture of observations and simulated samples.

For $\tilde{x}=\left({\tilde{x}}_{1},\cdots ,{\tilde{x}}_{N}\right)\in R^{n_{1}}\times \cdots \times R^{n_{N}}$ and $\tilde{y}=\left({\tilde{y}}_{1},\cdots ,{\tilde{y}}_{N}\right)\in R^{n_{1}}\times \cdots \times R^{n_{N}}$, $\tilde{x}\leq \tilde{y}$ means $x_{k}^{i,j}\leq y_{k}^{i,j},i=1,\cdots ,N,j=1,\cdots ,K,k=1,\cdots ,n_{ij}$. Denote $\tilde{1}:=\left({\tilde{1}}_{1},\cdots ,{\tilde{1}}_{N}\right)\in R^{n_{1}}\times \cdots \times R^{n_{N}}$, where ${\tilde{1}}_{i}:=\left(1,\cdots ,1\right)\in R^{n_{i}}$ for i=1,⋯,N; $\tilde{0}:=\left({\tilde{0}}_{1},\cdots ,{\tilde{0}}_{N}\right)\in R^{n_{1}}\times \cdots \times R^{n_{N}}$, where ${\tilde{0}}_{i}:=\left(0,\cdots ,0\right)\in R^{n_{i}}$ for i=1,⋯,N. ${\tilde{e}}^{i}:=\left({\tilde{0}}_{1},\cdots ,{\tilde{0}}_{i-1},{\tilde{1}}_{i},{\tilde{0}}_{i+1},\cdots ,{\tilde{0}}_{N}\right)\in R^{n_{1}}\times \cdots \times R^{n_{N}}$ for i=1,⋯,N. ${\tilde{e}}_{k}^{i,j}:=\left(0,\cdots ,0,1,0,\cdots ,0\right)\in R^{n}$ where 1 is located in the $\left(\sum_{s=1}^{i-1}n_{s}+\sum_{m=1}^{j-1}n_{im}+k\right)$th position, and ${\tilde{e}}_{1}^{1,1},\cdots ,{\tilde{e}}_{n_{NK}}^{N,K}$ are the canonical basis of $R^{n}$. 〈·,·〉 denotes the usual inner product on the Euclidean space. Given a set *A*, int(A) means the interior point of A, co(A) means the convex hull of A and cl(A) the closure of A. Denote

$$W:=\left\{\tilde{\omega }=\left({\tilde{\omega }}_{1},\cdots ,{\tilde{\omega }}_{N}\right)\in R^{n_{1}}\times \cdots \times R^{n_{N}}:\tilde{\omega }\geq 0,\langle {\tilde{1}}_{i},{\tilde{\omega }}_{i}\rangle =1,i=1,\cdots ,N\right\}.$$

We begin with the acceptance set. Given a nonempty set $A\subset R^{n}$, we list only some of its axioms, which will be closely related to the present paper, as follows.

(A1) Finiteness: $\sup\{\sum_{i=1}^{N}m_{i}:\left(m_{1}{\tilde{1}}_{1},\cdots ,m_{N}{\tilde{1}}_{N}\right)\in A\}<+\infty$.
(A2) Monotonicity: For any $\tilde{x}\in A,\tilde{y}\in R^{n}$, if $\tilde{y}\leq \tilde{x}$, then $\tilde{y}\in A$.
(A3) Convexity: A is a convex set.
(A4) Cone: A is a positively homogeneous cone, that is, $\lambda \tilde{x}\in A$ for any λ>0 and $\tilde{x}\in A$.

The interpretations of the axioms (A1)–(A4) are as follows. The finiteness means that the maximum of the possible deterministic losses of the portfolio should not be infinity. The monotonicity implies that if a portfolio with large losses is accepted, then a portfolio with less losses should also be accepted. The convexity means that given two accepted portfolios, any convex combination of these two portfolios should also be accepted. The axiom of cone means that given an accepted portfolio, any multiple of the portfolio should also be accepted.

**Definition** **1.** *A nonempty subset* A *of* $R^{n}$ *is called an acceptance set if it satisfies axioms A1–A2, and called a convex acceptance set if it satisfies axioms A1–A3, and called a coherent acceptance set if it satisfies axioms A1–A4.*

Next, we list some axioms for a mapping $\rho :R^{n}\to R$ as follows, which were proposed by [23], see also [19,20,21].

(B1) Translation invariance: For any $\tilde{x}\in R^{n},a_{i}\in R,i=1,\cdots ,N$,
  $$\rho \left(\tilde{x}+\left(a_{1}{\tilde{1}}_{1},\cdots ,a_{N}{\tilde{1}}_{N}\right)\right)=\rho \left(\tilde{x}\right)+\sum_{i=1}^{N}a_{i}.$$
(B2) Monotonicity: For any $\tilde{x},\tilde{y}\in R^{n}$, if $\tilde{x}\leq \tilde{y}$, then $\rho \left(\tilde{x}\right)\leq \rho \left(\tilde{y}\right)$.
(B3) Convexity: For any $\tilde{x},\tilde{y}\in R^{n}$ and α∈[0,1],
  $$\begin{array}{c} \rho \left(\alpha \tilde{x}+\left(1-\alpha \right)\tilde{y}\right)\leq \alpha \rho \left(\tilde{x}\right)+\left(1-\alpha \right)\rho \left(\tilde{y}\right). \end{array}$$
(B4) Positive homogeneity: For any $\tilde{x}\in R^{n}$ and λ>0, $\rho \left(\lambda \tilde{x}\right)=\lambda \rho \left(\tilde{x}\right)$.

The interpretations of the axioms (B1)–(B4) are as follows. The translation invariance says that adding the sure loss $\sum_{i=1}^{N}a_{i}$ to a portfolio $\tilde{x}$ simply increases the risk by $\sum_{i=1}^{N}a_{i}$. Monotonicity means that the larger loss of the portfolio is, the more risky it is. Convexity means that diversification does not increase the risk, that is, the risk of a diversified position $\alpha \tilde{x}+\left(1-\alpha \right)\tilde{y}$ is less or equal to the weighted average of the individual risks. Positive homogeneity means that when the loss of a portfolio increases (or decreases) linearly with a multiple, then the risk of the portfolio should also increase (or decreases) linearly with the same multiple.

On a general level, a multivariate risk statistic ρ is any mapping from $R^{n}$ to the real numbers R. It is a data-based version of a multivariate risk measure, and assigns $\left({\tilde{x}}_{1},\cdots ,{\tilde{x}}_{N}\right)$, the data representation of the random losses $\left(X_{1},\cdots ,X_{N}\right)$, a real number $\rho (\left({\tilde{x}}_{1},\cdots ,{\tilde{x}}_{N}\right))$, the risk measurement of $\left(X_{1},\cdots ,X_{N}\right)$.

**Definition** **2.** *A mapping* $\rho :R^{n}\to R$ *is called a multivariate monetary risk statistic if ρ satisfies axioms B1–B2, and called a multivariate convex risk statistic if it satisfies axioms B1–B3, and called a multivariate coherent risk statistic if it satisfies axioms B1–B4.*

Given an acceptance set A, we say that $\tilde{x}\in R^{n}$ is acceptable (with respect to A) if $\tilde{x}\in A$. For a position $\tilde{X}$, represented by its sample data $\tilde{x}=\left({\tilde{x}}_{1},\cdots ,{\tilde{x}}_{N}\right)\in R^{n_{1}}\times \cdots \times R^{n_{N}}$, define the capital requirement $\rho_{A}\left(\tilde{x}\right)$ for $\tilde{x}$ as the minimal amount $\sum_{i=1}^{N}m_{i}$ such that $\left({\tilde{x}}_{1}-m_{1}{\tilde{1}}_{1},\cdots ,{\tilde{x}}_{N}-m_{N}{\tilde{1}}_{N}\right)$ is acceptable by A. That is,

(1) $$\begin{array}{c} \rho_{A}\left(\tilde{x}\right):=\inf\left\{\sum_{i=1}^{N}m_{i}:\left({\tilde{x}}_{1}-m_{1}{\tilde{1}}_{1},\cdots ,{\tilde{x}}_{N}-m_{N}{\tilde{1}}_{N}\right)\in A,m_{i}\in R,1\leq i\leq N\right\} \end{array}$$

for any $\tilde{x}=\left({\tilde{x}}_{1},\cdots ,{\tilde{x}}_{N}\right)\in R^{n_{1}}\times \cdots \times R^{n_{N}}$. we call $\rho_{A}$ the risk statistic induced by A.

On the other hand, for any multivariate monetary risk statistic $\rho :R^{n}\to R$, we can define a set

(2) $$\begin{array}{c} A_{\rho }:=\left\{\tilde{x}\in R^{n}:\rho \left(\tilde{x}\right)\leq 0\right\}. \end{array}$$

Note that Proposition 2 below will show that $A_{\rho }$ satisfies axioms (A1)–(A2), i.e., $A_{\rho }$ is an acceptance set, and we call the set $A_{\rho }$ the acceptance set of ρ.

The following two propositions discuss the properties of $\rho_{A}$ and $A_{\rho }$ for given acceptance set A and risk statistics ρ, respectively. Their proofs will be postponed to Section 4.

**Proposition** **1.** *Let* $A\subset R^{n}$ *be an acceptance set and* $\rho_{A}$ *be defined by Equation (1). Then,*

*(1)*  $\rho_{A}$ *is a multivariate monetary risk statistic.*
*(2)*  $\rho_{A}$ *italicis a multivariate convex risk statistic if* A *is convex.*
*(3)*  $\rho_{A}$ *is a multivariate coherent risk statistic if* A *is a convex cone.*
*(4)*  A *is a subset of* $A_{\rho_{A}}$ *, and* $\mathrm{cl}\left(A\right)=A_{\rho_{A}}$ *if* A *satisfies the finiteness and monotonicity axioms.*

**Proposition** **2.** *Let* $\rho :R^{n}\to R$ *be a multivariate monetary risk statistic and* $A_{\rho }$ *be defined by Equation (2). Then,*

*(1)*  $A_{\rho }$ *is an acceptance set.*
*(2)*  *ρ can be recovered from* $A_{\rho }$ *, i.e.,* $\rho =\rho_{A_{\rho }}$ *.*
*(3)*  $A_{\rho }$ *is a convex acceptance set if and only if ρ is convex.*
*(4)*  $A_{\rho }$ *is a coherent acceptance set if and only if ρ is coherent.*

The following representation result for multivariate convex risk statistics has been provided in [23].

**Lemma** **1.** *A mapping* $\rho :R^{n}\to R$ *is a multivariate convex risk statistic if and only if there exists a set of weights* $\tilde{W}\subseteq W$ *such that*

$$\rho \left(\tilde{x}\right)=\underset{\tilde{\omega }\in \tilde{W}}{\sup}\left\{\langle \tilde{x},\tilde{\omega }\rangle -\alpha_{\min}\left(\tilde{\omega }\right)\right\},$$

*for any* $\tilde{x}\in R^{n}$ *, where the penalty function* $\alpha_{\min}:R^{n}\to R$ *is given by*

$$\alpha_{\min}\left(\tilde{\omega }\right):=\underset{\tilde{x}\in A_{\rho }}{\sup}\left\{\langle \tilde{x},\tilde{\omega }\rangle \right\},$$

*where* $A_{\rho }:=\left\{\tilde{x}\in R^{n}:\;\rho \left(\tilde{x}\right)\leq 0\right\}$ *.*

**Definition** **3.** *A function* ℓ:R→R *is called a loss function, if it is increasing and not identically constant.*

The conjugate function of a proper convex function ℓ:R→R is defined as

$$\ell^{*}\left(y\right):=\underset{x\in R}{\sup}\left\{xy-\ell \left(x\right)\right\},\;y\in R.$$

The following two lemmas characterize the conjugate functions of loss functions, which were provided by [24] and will be used to prove the main results.

**Lemma** **2.** *Let* ${\left\{\ell^{n}\right\}}_{n\in N}$ *be a sequence of convex loss functions which decreases pointwise to the convex loss function ℓ, then the corresponding conjugate functions* ${\left(\ell^{n}\right)}^{*}$ *increase pointwise to* $\ell^{*}$ *.*

**Lemma** **3.** *Let ℓ be a convex loss function, then the conjugate funcion* $\ell^{*}$ *has the following properties.*

*(1)*  $\ell^{*}\left(0\right)=-\inf_{x\in R}\ell \left(x\right)$ *and* $\ell^{*}\left(y\right)\geq -\ell \left(0\right)$ *for any* y∈R *.*
*(2)*  $\frac{\ell^{*}\left(y\right)}{y}\to \infty$ *as* y↑∞ *.*
*(3)*  *Denote by* $J:={\left(\ell^{*}\right)}^{\prime }$ *, the derivative function of* $\ell^{*}$ *, then for any* x,y∈R *,*
  (3) $$\begin{array}{c} xy\leq \ell \left(x\right)+\ell^{*}\left(y\right)\;\mathrm{with}\;\mathrm{equality}\;\mathrm{if}\;x=J\left(y\right). \end{array}$$

For now on, let the loss functions $\ell_{i},\cdots ,\ell_{N}$ be convex. For each i=1,⋯,N, let $z_{0,i}$ be an interior point of the range of $\ell_{i}$. Denote

$$\begin{array}{c} z_{0}:=z_{0,1}+\cdots +z_{0,N}. \end{array}$$

We define the following acceptance set,

(4) $$\begin{array}{c} B:=\left\{\tilde{x}\in R^{n}:\sum_{i=1}^{N}\frac{1}{n_{i}}\sum_{j=1}^{K}\sum_{k=1}^{n_{ij}}\ell_{i}\left(x_{k}^{i,j}\right)\leq z_{0}\right\}. \end{array}$$

The interpretation of $z_{0,i}$ and the acceptance set B as in Equation (4) are as follows. $z_{0,i}$ denotes the tolerance level towards loss of the *i*th financial positions at which the investor can bear. Therefore, $z_{0}=z_{0,1}+\cdots +z_{0,N}$ denotes the tolerance level towards loss of the portfolio $\tilde{X}=\left(X_{1},\cdots ,X_{N}\right)$ at which the investor can bear. For example, for risk-averse investor, the tolerance levels $z_{0,i}$’s could be chosen as zero provided that the zero is an interior point of the range of $\ell_{i}$. For a risk-neutral investor, the tolerance levels $z_{0,i}$’s could be chosen as a positive but small numbers. For a risk-appetitive investor, the tolerance levels $z_{0,i}$’s could be chosen as certain large positive numbers. Therefore, these three situations can also be regarded as kinds of trading preferences for an investor. Note that given the tolerance levels $z_{0,i}$’s by the investor, not all of financial positions are in the acceptance set B.

Note that the acceptance set B defined as Equation (4) is convex and closed, as each loss function $\ell_{i}$ is convex, 1≤i≤N. Next, according to the acceptance set B, we can introduce the definition of the multivariate shortfall risk statistics.

**Definition** **4.** *The mapping* $\rho_{B}:R^{n_{1}}\times \cdots \times R^{n_{N}}\to R$ *defined by*

(5) $$\begin{array}{c} \rho_{B}\left(\tilde{x}\right):=\inf\left\{\sum_{i=1}^{N}m_{i}:\left({\tilde{x}}_{1}-m_{1}{\tilde{1}}_{1},\cdots ,{\tilde{x}}_{N}-m_{N}{\tilde{1}}_{N}\right)\in B\right\} \end{array}$$

*for* $\tilde{x}=\left({\tilde{x}}_{1},\cdots ,{\tilde{x}}_{N}\right)\in R^{n_{1}}\times \cdots \times R^{n_{N}}$ *, is called a multivariate shortfall risk statistic.*

By the definition of the acceptance set B in Equation (4), the multivariate shortfall risk statistic $\rho_{B}$ can be rewritten as

$$\begin{array}{c} \rho_{B}\left(\tilde{x}\right)=\inf\left\{\sum_{i=1}^{N}m_{i}:\sum_{i=1}^{N}\frac{1}{n_{i}}\sum_{j=1}^{K}\sum_{k=1}^{n_{ij}}\ell_{i}\left(x_{k}^{i,j}-m_{i}\right)\leq z_{0}\right\} \end{array}$$

for $\tilde{x}=\left({\tilde{x}}_{1},\cdots ,{\tilde{x}}_{N}\right)=\left(x_{1}^{1,1},\cdots ,x_{n_{11}}^{1,1},\cdots ,x_{1}^{1,K},\cdots ,x_{n_{1k}}^{1,K},\cdots ,x_{1}^{N,1},\cdots ,x_{n_{N1}}^{N,1},\cdots ,x_{1}^{N,K},\cdots ,x_{n_{NK}}^{N,K}\right)\in R^{n_{1}}\times \cdots \times R^{n_{N}}$.

**Remark** **1.** *As each convex loss function* $\ell_{i}$ *is convex,* 1≤i≤N *, it is not hard to verify that* B *satisfies A1–A3. Therefore, a multivariate shortfall risk statistic* $\rho_{B}$ *is also a multivariate convex risk statistic. By Proposition 1,* $B=A_{\rho_{B}}$ *, as* B *is closed.*

Now, we introduce the definition of multivariate divergence risk statistics.

**Definition** **5.** *For each* i=1,⋯,N *, let* $g_{i}:\left[0,+\infty \right)\to R\cup \left\{+\infty \right\}$ *be a lower semicontinuous convex function satisfying* $g_{i}\left(1\right)<\infty$ *and the superlinear growth condition* $\frac{g_{i}\left(x\right)}{x}\to +\infty$ *as* x↑∞ *. The mapping* $I_{\left(g_{1},\cdots ,g_{N}\right)}:{\left[0,+\infty \right)}^{n_{1}}\times \cdots \times {\left[0,+\infty \right)}^{n_{N}}\to R$ *defined by*

$$\begin{array}{c} I_{\left(g_{1},\cdots ,g_{N}\right)}\left(\tilde{\omega }\right):=\sum_{i=1}^{N}\frac{1}{n_{i}}\sum_{j=1}^{K}\sum_{k=1}^{n_{ij}}g_{i}\left(n_{i}\omega_{k}^{i,j}\right), \end{array}$$

*for* $\tilde{\omega }=\left({\tilde{\omega }}_{1},\cdots ,{\tilde{\omega }}_{N}\right)=\left(\omega_{1}^{1,1},\cdots ,x_{n_{11}}^{1,1},\cdots ,\omega_{1}^{1,K},\cdots ,\omega_{n_{1k}}^{1,K},\cdots ,\omega_{1}^{N,1},\cdots ,\omega_{n_{N1}}^{N,1},\cdots ,\omega_{1}^{N,K},\cdots ,\omega_{n_{NK}}^{N,K}\right)\in {\left[0,+\infty \right)}^{n_{1}}\times \cdots \times {\left[0,+\infty \right)}^{n_{N}}$ *, is called a multivariate* $(g_{1},\cdots ,g_{N})-$ *divergence function.*

*The mapping* $\rho_{\left(g_{1},\cdots ,g_{N}\right)}:R^{n_{1}}\times \cdots \times R^{n_{N}}\to R$ *defined by*

$$\begin{array}{c} \rho_{\left(g_{1},\cdots ,g_{N}\right)}\left(\tilde{x}\right):=\underset{\tilde{\omega }\in W}{\sup}\left\{\sum_{i=1}^{N}\langle {\tilde{x}}_{i},{\tilde{\omega }}_{i}\rangle -I_{\left(g_{1},\cdots ,g_{N}\right)}\left(\tilde{\omega }\right)\right\}, \end{array}$$

*for* $\tilde{x}=\left({\tilde{x}}_{1},\cdots ,{\tilde{x}}_{N}\right)\in R^{n_{1}}\times \cdots \times R^{n_{N}}$ *, is called a multivariate divergence risk statistic.*

**Remark** **2.** 

*(1)*  *If* $g_{i}:\left[0,+\infty \right)\to R\cup \left\{+\infty \right\}$ *is a convex function for each* 1≤i≤N *, then* $\left(\beta ,\omega \right)\mapsto \beta g_{i}\left(\frac{\omega }{\beta }\right)$ *is a convex function on* (0,+∞)×[0,+∞),i=1,⋯,N *.*
*(2)*  *For any* i=1,⋯,N *, let* $g_{i}:\left[0,+\infty \right)\to R\cup \left\{+\infty \right\}$ *be a lower semicontinuous convex function satisfying* $g_{i}\left(1\right)<\infty$ *and the superlinear growth condition* $\frac{g_{i}\left(x\right)}{x}\to +\infty$ *as* x↑∞ *. For* β>0 *, denote* ${\tilde{g}}_{i}\left(\beta ,x\right):=\beta g_{i}\left(\frac{x}{\beta }\right)$ *, the corresponding multivariate* $({\tilde{g}}_{1},\cdots ,{\tilde{g}}_{N})-$ *divergence function*
  $$\begin{array}{c} I_{\left({\tilde{g}}_{1},\cdots ,{\tilde{g}}_{N}\right)}\left(\beta ,\tilde{\omega }\right):=\sum_{i=1}^{N}\frac{1}{n_{i}}\sum_{j=1}^{K}\sum_{k=1}^{n_{ij}}\beta g_{i}\left(\frac{n_{i}\omega_{k}^{i,j}}{\beta }\right) \end{array}$$
  *is a convex function on* $\left(0,+\infty \right)\times {\left[0,+\infty \right)}^{n}$ *. For any fixed* $\tilde{x}\in R^{n_{1}}\times \cdots \times R^{n_{N}}$ *, define*
  $$G\left(\beta \right):=G_{\tilde{x}}\left(\beta \right):=\left\{\begin{array}{cc} -\rho_{\left({\tilde{g}}_{1},\cdots ,{\tilde{g}}_{N}\right)}\left(\tilde{x}\right)=\underset{\tilde{\omega }\in W}{\inf}\left\{-\langle \tilde{x},\tilde{\omega }\rangle +I_{\left({\tilde{g}}_{1},\cdots ,{\tilde{g}}_{N}\right)}\left(\beta ,\tilde{\omega }\right)\right\}\; & \mathrm{if}\;\beta >0,\\ +\infty & \mathrm{otherwise}, \end{array}\right.$$
  *it is not hard to check that* G(β) *is a lower semicontinuouse convex function on* (0,+∞) *.*

## 3. Main Results

In this section, we state the main results of this paper, and their proofs will be postponed to the next section. Specifically, we will study the properties and the representation results for multivariate shortfall risk statistics and divergence risk statistics.

The following proposition shows that $\rho_{B}$ is a multivariate convex risk statistics, which can be steadily implied by Proposition 1, and therefore we omit its proof here.

**Proposition** **3.** *The multivariate shortfall risk statistic* $\rho_{B}:R^{n}\to R$ *satisfies the translation invariance, monotonicity, and convexity.*

Now, we are ready to state the first main result of the present paper, which provides the representation results for multivariate shortfall risk statistics.

**Theorem** **1.** *Let* $\rho_{B}:R^{n}\to R$ *be a multivariate shortfall risk statistic associated with the convex loss functions* $\left(\ell_{1},\cdots ,\ell_{N}\right)$ *and* $z_{0}$ *. Then the minimal penalty function of* $\rho_{B}$ *is given by*

(6) $$\begin{array}{c} \alpha_{min}\left(\tilde{\omega }\right)=\underset{\lambda >0}{\inf}\frac{1}{\lambda }\left\{z_{0}+\sum_{i=1}^{N}\frac{1}{n_{i}}\sum_{j=1}^{K}\sum_{k=1}^{n_{ij}}\ell_{i}^{*}\left(\lambda n_{i}\omega_{k}^{i,j}\right)\right\},\;\;\tilde{\omega }\in W, \end{array}$$

*where* $\ell_{i}^{*}$ *is the conjugate function of* $\ell_{i}$ *. In particular, for any* $\tilde{x}\in R^{n}$ *,*

$$\begin{array}{c} \rho_{B}\left(\tilde{x}\right)=\underset{\tilde{\omega }\in W}{\sup}\left\{\langle \tilde{x},\tilde{\omega }\rangle -\underset{\lambda >0}{\inf}\frac{1}{\lambda }\left\{z_{0}+\sum_{i=1}^{N}\frac{1}{n_{i}}\sum_{j=1}^{K}\sum_{k=1}^{n_{ij}}\ell_{i}^{*}\left(\lambda n_{i}\omega_{k}^{i,j}\right)\right\}\right\}. \end{array}$$

Theorem 1 shows that given *N* convex loss functions $\ell_{1},\cdots ,\ell_{N}$ and the corresponding totally average loss level $z_{0}$, the minimal penalty function $\alpha_{\min}$ can be expressed in terms of $\ell_{1},\cdots ,\ell_{N}$. From a practical point of view, an investor may have uncertainty about kinds of loss function (i.e., the utility function). Next, we will discuss this issue in the case where N=1, and will provide a slight more general result, which is similar to Theorem 1.

Let L be a class of convex loss functions. Given ℓ∈L, similar to Equation (4), we can define the corresponding average loss level $z_{0}=z_{0}\left(\ell \right)$ and the acceptance set $B=B(\ell ,z_{0})$ by

$$\begin{array}{c} B\left(\ell ,z_{0}\right):=\left\{\tilde{x}\in R^{n}:\frac{1}{n}\sum_{j=1}^{K}\sum_{k=1}^{n_{j}}\ell \left(x_{jk}\right)\leq z_{0}\right\}. \end{array}$$

Assume that

(7) $$\begin{array}{c} B_{L}:=\underset{\ell \in L}{⋂}B\left(\ell ,z_{0}\right)\neq \emptyset . \end{array}$$

Note that $B_{L}$ is convex and closed. Therefore, similar to Equation (5), we can define a convex risk statistic $\rho_{L}:=\rho_{B_{L}}:R^{n}\to R$ by

(8) $$\begin{array}{c} \rho_{L}\left(\tilde{x}\right):=\inf\left\{m\in R:\tilde{x}-m\tilde{1}\in B_{L}\right\}. \end{array}$$

Theorem 1 yields the following representation for the convex risk statistic $\rho_{L}$.

**Corollary** **1.** *Assume that* $B_{L}$ *defined by Equation (7) is not empty. Then the multivariate convex risk statistic* $\rho_{L}$ *defined by Equation (8) can be expressed by*

$$\begin{array}{c} \rho_{L}\left(\tilde{x}\right)=\underset{\tilde{\omega }\in \tilde{W}p}{\sup}\left\{\langle \tilde{x},\tilde{\omega }\rangle -\alpha_{\min}\left(\tilde{\omega }\right)\right\}, \end{array}$$

*where the minimal penalty function* $\alpha_{\min}:R^{n}\to R$ *is given by*

$$\begin{array}{c} \alpha_{\min}\left(\tilde{\omega }\right)=\underset{\lambda >0}{\inf}\underset{\ell \in L}{\inf}\left\{\frac{z_{0}\left(\ell \right)}{\lambda }+\frac{1}{\lambda n}\sum_{j=1}^{K}\sum_{k=1}^{n_{j}}\ell^{*}\left(\lambda n\omega_{jk}\right)\right\},\;\tilde{\omega }\in \tilde{W}, \end{array}$$

*where* $\tilde{W}=\left\{\tilde{\omega }=\left(\omega_{1},\cdots ,\omega_{n}\right)\in R^{n}:\tilde{\omega }\geq 0,\sum_{i=1}^{n}\omega_{i}=1\right\}$ *.*

**Remark** **3.** *Corollary 1 can be considered as the data-based (or empirical) version of Föllmer and Schied (2011, Corollary 4.119).*

Next, we will state the second main result of the present paper, which characterizes the (univariate) shortfall risk statistic $\rho_{B}$ defined by Equation (5), being coherent by means of the loss function *ℓ* in a special case where N=1.

**Theorem** **2.** *Assume that* $\rho_{B}:R^{n}\to R$ *is a univariate shortfall risk statistic associated with a convex loss function ℓ and* $z_{0}\in \mathrm{int}\left\{\ell \left(y\right):y\in R\right\}$ *, and that* $\inf_{x\in R}\ell \left(x\right)=-\infty$ *and* $\sup_{x\in R}\ell \left(x\right)=+\infty$ *. Then* $\rho_{B}$ *is coherent if and only if* $\ell \left(x\right)=z_{0}+\alpha x^{+}-\beta x^{-}$ *with* 0<β≤α *.*

Finally, we will state the last main result of the present paper, which provides the representation result for multivariate divergence risk statistics.

**Theorem** **3.** *Let* $g_{i}:\left[0,+\infty \right)\to R\cup \left\{+\infty \right\}$ *be a lower semicontinuous convex function satisfying* $g_{i}\left(1\right)<\infty$ *and the superlinear growth condition* $\frac{g_{i}\left(x\right)}{x}\to +\infty$ *as* x↑∞ *for each* 1≤i≤N *; then, the multivariate divergence risk statistic* $\rho_{\left(g_{1},\cdots ,g_{N}\right)}$ *is of the following expression*

(9) $$\begin{array}{c} \rho_{\left(g_{1},\cdots ,g_{N}\right)}\left(\tilde{x}\right)=\underset{\left(z_{1},\cdots ,z_{N}\right)\in R^{N}}{\inf}\left\{\sum_{i=1}^{N}\frac{1}{n_{i}}\sum_{j=1}^{K}\sum_{k=1}^{n_{ij}}g_{i}^{*}\left(x_{k}^{i,j}-z_{i}\right)+\sum_{i=1}^{N}z_{i}\right\}, \end{array}$$

*for* $\tilde{x}=\left({\tilde{x}}_{1},\cdots ,{\tilde{x}}_{N}\right)=\left(x_{1}^{1,1},\cdots ,x_{n_{11}}^{1,1},\cdots ,x_{1}^{1,K},\cdots ,x_{n_{1k}}^{1,K},\cdots ,x_{1}^{N,1},\cdots ,x_{n_{N1}}^{N,1},\cdots ,x_{1}^{N,K},\cdots ,x_{n_{NK}}^{N,K}\right)\in R^{n_{1}}\times \cdots \times R^{n_{N}}$ *, where* $g_{i}^{*}\left(y\right)$ *is the conjugate function of* $g_{i}$ *for each* 1≤i≤N *.*

## 4. Proofs of Main Results

In this section, we will provide an alternate proofs of Propositions 1 and 2, and all the proofs of the results stated in Section 3.

**Proof** **of** **Proposition** **1.** (1) Let A be an acceptance set, i.e., satisfying the finiteness and monotonicity, it is not hard to verify the translation invariance and monotonicity of $\rho_{A}$. Therefore, we only need to show that $\rho_{A}$ takes finite values.

Let $\tilde{x}=\left({\tilde{x}}_{1},\cdots ,{\tilde{x}}_{N}\right)\in A$ be fixed (as $\rho_{A}$ is nonempty, such $\tilde{x}$ must exist). Then we know that

$$\begin{array}{c} \tilde{0}\in \left\{\left(m_{1},\cdots ,m_{N}\right)\in R^{N}:\left({\tilde{x}}_{1}-m_{1}{\tilde{1}}_{1},\cdots ,{\tilde{x}}_{N}-m_{N}{\tilde{1}}_{N}\right)\in A\right\}, \end{array}$$

which, together with the definition of $\rho_{A}$, implies that $\rho_{A}\left(\tilde{x}\right)\leq 0$.

For any given $\tilde{y}=\left({\tilde{y}}_{1},\cdots ,{\tilde{y}}_{N}\right)\in R^{n_{1}}\times \cdots \times R^{n_{N}}$, clearly, there exists a $\tilde{m}=\left(m_{1},\cdots ,m_{N}\right)\in R^{N}$ such that $\left({\tilde{y}}_{1}-m_{1}{\tilde{1}}_{1},\cdots ,{\tilde{y}}_{N}-m_{N}{\tilde{1}}_{N}\right)\leq \left({\tilde{x}}_{1},\cdots ,{\tilde{x}}_{N}\right)$. Thus,

$$\begin{array}{c} \rho_{A}\left(\tilde{y}\right)-\sum_{i=1}^{N}m_{i}=\rho_{A}\left(\tilde{x}+\left(m_{1}{\tilde{1}}_{1},\cdots ,m_{N}{\tilde{1}}_{N}\right)\right)\leq \rho_{A}\left(\tilde{x}\right)\leq 0, \end{array}$$

which yields that $\rho_{A}\left(\tilde{y}\right)\leq \sum_{i=1}^{N}m_{i}<+\infty$.

On the other hand, by the finiteness of A, we have that

$$\begin{array}{cc} \rho_{A}\left(\tilde{0}\right) & =\inf\left\{\sum_{i=1}^{N}m_{i}:\left(0-m_{1}{\tilde{1}}_{1},\cdots ,0-m_{N}{\tilde{1}}_{N}\right)\in A\right\}\\ \; & =-\sup\left\{\sum_{i=1}^{N}m_{i}:\left(m_{1}{\tilde{1}}_{1},\cdots ,m_{N}{\tilde{1}}_{N}\right)\in A\right\}\\ \; & >-\infty . \end{array}$$

Clearly, there exists a $\tilde{m^{\prime }}=\left(m_{1}^{\prime },\cdots ,m_{N}^{\prime }\right)\in R^{N}$ such that $({\tilde{y}}_{1}-m_{1}^{\prime }{\tilde{1}}_{1},\cdots ,{\tilde{y}}_{N}-m_{N}^{\prime }{\tilde{1}}_{N})\geq 0$. Therefore, from the translation invariance and monotonicity of $\rho_{A}$, it follows that

$$\begin{array}{c} \rho_{A}\left(\tilde{y}\right)\geq \rho_{A}\left(\tilde{0}\right)+\sum_{i=1}^{N}m_{i}^{\prime }>-\infty . \end{array}$$

(2) Let A be a convex acceptance set. Then by part (1) above, we only need to show the convexity of $\rho_{A}$.

Let $\tilde{x}=\left({\tilde{x}}_{1},\cdots ,{\tilde{x}}_{N}\right),\tilde{y}=\left({\tilde{y}}_{1},\cdots ,{\tilde{y}}_{N}\right)\in R^{n_{1}}\times \cdots \times R^{n_{N}}$. For any $(a_{1},\cdots ,a_{N}),$$(b_{1},$⋯,$b_{N})$$\in R^{N}$ with $\left({\tilde{x}}_{1}-a_{1}{\tilde{1}}_{1},\cdots ,{\tilde{x}}_{N}-a_{N}{\tilde{1}}_{N}\right),\left({\tilde{y}}_{1}-b_{1}{\tilde{1}}_{1},\cdots ,{\tilde{y}}_{N}-b_{N}{\tilde{1}}_{N}\right)\in A$, and any α∈[0,1], from the convexity of A, it follows that

$$\begin{array}{c} \alpha \left({\tilde{x}}_{1}-a_{1}{\tilde{1}}_{1},\cdots ,{\tilde{x}}_{N}-a_{N}{\tilde{1}}_{N}\right)+\left(1-\alpha \right)\left({\tilde{y}}_{1}-b_{1}{\tilde{1}}_{1},\cdots ,{\tilde{y}}_{N}-b_{N}{\tilde{1}}_{N}\right)\in A, \end{array}$$

which, together with the definition of $\rho_{A}$, yields that

$$\begin{array}{c} \rho_{A}\left(\alpha \left({\tilde{x}}_{1}-a_{1}{\tilde{1}}_{1},\cdots ,{\tilde{x}}_{N}-a_{N}{\tilde{1}}_{N}\right)+\left(1-\alpha \right)\left({\tilde{y}}_{1}-b_{1}{\tilde{1}}_{1},\cdots ,{\tilde{y}}_{N}-b_{N}{\tilde{1}}_{N}\right)\right)\leq 0. \end{array}$$

Therefore, by the translation invariance of $\rho_{A}$,

$$\begin{array}{cc} 0 & \geq \rho_{A}\left[\alpha \left({\tilde{x}}_{1}-a_{1}{\tilde{1}}_{1},\cdots ,{\tilde{x}}_{N}-a_{N}{\tilde{1}}_{N}\right)+\left(1-\alpha \right)\left({\tilde{y}}_{1}-b_{1}{\tilde{1}}_{1},\cdots ,{\tilde{y}}_{N}-b_{N}{\tilde{1}}_{N}\right)\right]\\ \; & =\rho_{A}\left[\alpha \left({\tilde{x}}_{1},\cdots ,{\tilde{x}}_{N}\right)+\left(1-\alpha \right)\left({\tilde{y}}_{1},\cdots ,{\tilde{y}}_{N}\right)\right]-\left[\alpha \sum_{i=1}^{N}a_{i}+\left(1-\alpha \right)\sum_{i=1}^{N}b_{i}\right], \end{array}$$

which implies the convexity of $\rho_{A}$ by letting $\sum_{i=1}^{N}a_{i}$ and $\sum_{i=1}^{N}b_{i}$ converge to $\rho_{A}\left(\tilde{x}\right)$ and $\rho_{A}\left(\tilde{y}\right)$, respectively.

(3) Let A be a coherent acceptance set. Then by parts (1) and (2) above, we only need to show the positive homogeneity of $\rho_{A}$.

For any λ>0 and $\tilde{x}=\left({\tilde{x}}_{1},\cdots ,{\tilde{x}}_{N}\right)\in R^{n_{1}}\times \cdots \times R^{n_{N}}$,

$$\begin{array}{cc} \rho_{A}\left(\lambda \tilde{x}\right)\; & =\inf\left\{\sum_{i=1}^{N}m_{i}:\left(\lambda {\tilde{x}}_{1}-m_{1}{\tilde{1}}_{1},\cdots ,\lambda {\tilde{x}}_{N}-m_{N}{\tilde{1}}_{N}\right)\in A\right\}\\ \; & =\inf\left\{\sum_{i=1}^{N}m_{i}:\left({\tilde{x}}_{1}-\frac{m_{1}}{\lambda }{\tilde{1}}_{1},\cdots ,{\tilde{x}}_{N}-\frac{m_{N}}{\lambda }{\tilde{1}}_{N}\right)\in A\right\}\\ \; & =\inf\left\{\sum_{i=1}^{N}\lambda m_{i}^{\prime }:\left({\tilde{x}}_{1}-m_{1}^{\prime }{\tilde{1}}_{1},\cdots ,{\tilde{x}}_{N}-m_{N}^{\prime }{\tilde{1}}_{N}\right)\in A\right\}\\ \; & =\lambda \inf\left\{\sum_{i=1}^{N}m_{i}^{\prime }:\left({\tilde{x}}_{1}-m_{1}^{\prime }{\tilde{1}}_{1},\cdots ,{\tilde{x}}_{N}-m_{N}^{\prime }{\tilde{1}}_{N}\right)\in A\right\}\\ \; & =\lambda \rho_{A}\left(\tilde{x}\right). \end{array}$$

(4) If $\tilde{x}\in A$, then $\rho_{A}\left(\tilde{x}\right)\leq 0$, which, together with the definition of $A_{\rho_{A}}$, yields that $\tilde{x}\in A_{\rho_{A}}$, and therefore $A\subseteq A_{\rho_{A}}$.

For any $\tilde{x}=\left({\tilde{x}}_{1},\cdots ,{\tilde{x}}_{N}\right),\tilde{y}=\left({\tilde{y}}_{1},\cdots ,{\tilde{y}}_{N}\right)\in R^{n_{1}}\times \cdots \times R^{n_{N}}$, denote $∥{\tilde{x}}_{i}{∥}_{\infty }:=\max\left\{x_{k}^{i,j},1\leq j\leq K,1\leq k\leq n_{ij}\right\}$ and $∥\tilde{x}∥:=\sum_{i=1}^{N}{∥{\tilde{x}}_{i}∥}_{\infty }$. Since clA is the closure of A in $R^{n}$, then clA is ∥·∥-closed. Note that

$$\begin{array}{c} \tilde{x}\leq \tilde{y}+(∥{\tilde{x}}_{1}-{\tilde{y}}_{1}{∥}_{\infty }{\tilde{1}}_{1},\cdots ,∥{\tilde{x}}_{N}-{\tilde{y}}_{N}{∥}_{\infty }{\tilde{1}}_{N}), \end{array}$$

which, along with the translation invariance and monotonicity of $\rho_{A}$, implies that

$$\begin{array}{c} \rho_{A}\left(\tilde{x}\right)\leq \rho_{A}\left(\tilde{y}\right)+\sum_{i=1}^{N}{∥{\tilde{x}}_{i}-{\tilde{y}}_{i}∥}_{\infty }. \end{array}$$

Reversing the roles of $\tilde{x}$ and $\tilde{y}$, we know that

(10) $$\begin{array}{c} |\rho_{A}\left(\tilde{x}\right)-\rho_{A}\left(\tilde{y}\right)|\leq \sum_{i=1}^{N}∥{\tilde{x}}_{i}-{\tilde{y}}_{i}{∥}_{\infty }=∥\tilde{x}-\tilde{y}∥. \end{array}$$

For any fixed $\tilde{x}\notin \mathrm{cl}\left(A\right)$, we can conclude that $\rho_{A}\left(\tilde{x}\right)>0$. In fact, take $m_{i}<-{∥{\tilde{x}}_{i}∥}_{\infty },1\leq i\leq N$, that is, $m_{i}{\tilde{1}}_{i}<-{∥{\tilde{x}}_{i}∥}_{\infty }{\tilde{1}}_{i}\leq {\tilde{x}}_{i}$ and $\sum_{i=1}^{N}m_{i}<-\sum_{i=1}^{N}∥{\tilde{x}}_{i}{∥}_{\infty }=-∥\tilde{x}∥$. As, cl(A) is ∥·∥-closed and $\tilde{x}\notin \mathrm{cl}\left(A\right)$, there is some λ∈(0,1), such that $\lambda \left(m_{1}{\tilde{1}}_{1},\cdots ,m_{N}{\tilde{1}}_{N}\right)+\left(1-\lambda \right)\tilde{x}\notin \mathrm{cl}\left(A\right)$. Therefore,

$$\begin{array}{c} 0<\rho_{A}\left(\lambda \left(m_{1}{\tilde{1}}_{1},\cdots ,m_{N}{\tilde{1}}_{N}\right)+\left(1-\lambda \right)\tilde{x}\right)=\rho_{A}\left(\left(1-\lambda \right)\tilde{x}\right)+\lambda \sum_{i=1}^{N}m_{i}. \end{array}$$

From Equation (10), it follows that

$$\begin{array}{c} |\rho_{A}\left(\tilde{x}\right)-\rho_{A}\left(\left(1-\lambda \right)\tilde{x}\right)|\leq \lambda ∥\tilde{x}∥. \end{array}$$

Therefore,

$$\begin{array}{c} \rho_{A}\left(\tilde{x}\right)\geq \rho_{A}\left(\left(1-\lambda \right)\tilde{x}\right)-\lambda ∥\tilde{x}∥>\lambda \left(-\sum_{i=1}^{N}m_{i}-∥\tilde{x}∥\right)>0. \end{array}$$

By the definition of $A_{\rho_{A}}$, we know that $\rho_{A}\left(\tilde{x}\right)>0$ implies $\tilde{x}\notin A_{\rho_{A}}$. Thus, $A_{\rho_{A}}\subseteq \mathrm{cl}\left(A\right)$. The proof of Proposition 1 is completed. □

**Proof** **of** **Proposition** **2.** (1) Let ρ be a multivariate monetary risk statistic, the monotonicity of $A_{\rho }$ is straightforward. If $\sup\{\sum_{i=1}^{N}m_{i}:\left(m_{1}{\tilde{1}}_{1},\cdots ,m_{N}{\tilde{1}}_{N}\right)\in A_{\rho }\}=+\infty$, then there exists a sequence ${\left\{\left(m_{1}^{k},\cdots ,m_{N}^{k}\right)\right\}}_{k\in N}$ where $\left(m_{1}^{k}{\tilde{1}}_{1},\cdots ,m_{N}^{k}{\tilde{1}}_{N}\right)\in A_{\rho }$ such that

$$\begin{array}{c} \lim_{k\to +\infty }\sum_{i=1}^{N}m_{i}^{k}=+\infty . \end{array}$$

By the translation invariance of ρ,

$$\begin{array}{c} \rho \left[\tilde{0}+\left(m_{1}^{k}{\tilde{1}}_{1},\cdots ,m_{N}^{k}{\tilde{1}}_{N}\right)\right]=\rho \left(\tilde{0}\right)+\sum_{i=1}^{N}m_{i}^{k}\to +\infty \;\left(k\to +\infty \right). \end{array}$$

This is a contradict with $\rho (\left(m_{1}^{k}{\tilde{1}}_{1},\cdots ,m_{N}^{k}{\tilde{1}}_{N}\right))\leq 0$.

(2) The translation invariance of ρ implies that for $\tilde{x}\in R^{n}$,

$$\begin{array}{cc} \rho_{A_{\rho }}\left(\tilde{x}\right) & =\inf\left\{\sum_{i=1}^{N}m_{i}:\tilde{x}-\left(m_{1}^{k}{\tilde{1}}_{1},\cdots ,m_{N}^{k}{\tilde{1}}_{N}\right)\in A_{\rho }\right\}\\ \; & =\inf\left\{\sum_{i=1}^{N}m_{i}:\rho \left(\tilde{x}-\left(m_{1}^{k}{\tilde{1}}_{1},\cdots ,m_{N}^{k}{\tilde{1}}_{N}\right)\right)\leq 0\right\}\\ \; & =\inf\left\{\sum_{i=1}^{N}m_{i}:\rho \left(\tilde{x}\right)\leq \sum_{i=1}^{N}m_{i}\right\}\\ \; & =\rho (\tilde{x}). \end{array}$$

(3) $A_{\rho }$ is a convex set if ρ is convex. The converse will follow from Proposition 1 together with $\rho_{A_{\rho }}=\rho$.

(4) By part (3) above, we only need to show the relationship between the cone of $A_{\rho }$ and the positive homogeneity of ρ. The cone of $A_{\rho }$ is straightforward from the positive homogeneity of ρ. The converse follows from $\rho_{A_{\rho }}=\rho$ and the part (3) in Proposition 1. The proof of Proposition 2 is completed. □

**Proof** **of** **Theorem** **1.** We will make full use of convex analysis to show the theorem.

Define a totally average loss function $L:R^{n}\to R$ as

$$\begin{array}{c} L\left(\tilde{x}\right):=\sum_{i=1}^{N}\frac{1}{n_{i}}\sum_{j=1}^{K}\sum_{k=1}^{n_{ij}}\ell_{i}\left(x_{k}^{i,j}\right) \end{array}$$

for any $\tilde{x}=\left({\tilde{x}}_{1},\cdots ,{\tilde{x}}_{N}\right)=(x_{1}^{1,1},\cdots ,x_{n_{11}}^{1,1},\cdots ,x_{1}^{1,K},\cdots ,x_{n_{1k}}^{1,K},\cdots ,x_{1}^{N,1},\cdots ,x_{n_{N1}}^{N,1},$⋯,$x_{1}^{N,K},$⋯,$x_{n_{NK}}^{N,K})$∈$R^{n_{1}}\times \cdots \times R^{n_{N}}$.

First, we show that it suffices to prove the claim for $L\left(\tilde{0}\right)<z_{0}$; otherwise, we can find some $\tilde{a}=\left({\tilde{a}}_{1},\cdots ,{\tilde{a}}_{N}\right)\in R^{n_{1}}\times \cdots \times R^{n_{N}}$ such that $L\left(\tilde{a}\right)<z_{0}$, as $z_{0}$ was assumed to be an interior point of $L(R^{n})$. Denote $b_{i}:=\min\left\{a_{k}^{i,j}:j=1,\cdots ,k;t=1,\cdots ,n_{ij}\right\},i=1,\cdots ,N,$ and $\tilde{b}:=\left(b_{1}{\tilde{1}}_{1},\cdots ,b_{N}{\tilde{1}}_{N}\right)$. Then, $\tilde{b}\leq \tilde{a}$, which, together with the increasing of *L*, yields that

$$\begin{array}{c} L\left(\tilde{b}\right)\leq L\left(\tilde{a}\right)<z_{0}. \end{array}$$

Let ${\tilde{\ell }}_{i}\left(x\right):=\ell_{i}\left(x+b_{i}\right),i=1,\cdots ,N$ define a function:

$$\begin{array}{c} \tilde{L}\left(\tilde{x}\right):=\sum_{i=1}^{N}\frac{1}{n_{i}}\sum_{j=1}^{K}\sum_{k=1}^{n_{ij}}{\tilde{\ell }}_{i}\left(x_{k}^{i,j}\right)=\sum_{i=1}^{N}\frac{1}{n_{i}}\sum_{j=1}^{K}\sum_{k=1}^{n_{ij}}\ell_{i}\left(x_{k}^{i,j}+b_{i}\right)=L\left(\tilde{x}+\tilde{b}\right) \end{array}$$

for any $\tilde{x}=\left({\tilde{x}}_{1},\cdots ,{\tilde{x}}_{N}\right)=(x_{1}^{1,1},\cdots ,x_{n_{11}}^{1,1},\cdots ,x_{1}^{1,K},\cdots ,x_{n_{1k}}^{1,K},\cdots ,x_{1}^{N,1},\cdots ,x_{n_{N1}}^{N,1},$⋯,$x_{1}^{N,K},$⋯,$x_{n_{NK}}^{N,K})$∈$R^{n_{1}}\times \cdots \times R^{n_{N}}$. It easy to verify that the function $\tilde{L}$ is increasing and satisfies the requirement $\tilde{L}\left(\tilde{0}\right)=L\left(\tilde{b}\right)<z_{0}$. From Equation (4), it follows that

$$\begin{array}{cc} \tilde{B}: & =\left\{\left({\tilde{x}}_{1},\cdots ,{\tilde{x}}_{N}\right)\in R^{n_{1}}\times \cdots \times R^{n_{N}}:\sum_{i=1}^{N}\frac{1}{n_{i}}\sum_{j=1}^{K}\sum_{k=1}^{n_{ij}}{\tilde{\ell }}_{i}\left(x_{k}^{i,j}\right)\leq z_{0}\right\}\\ \; & =\left\{\left({\tilde{x}}_{1},\cdots ,{\tilde{x}}_{N}\right)\in R^{n_{1}}\times \cdots \times R^{n_{N}}:\sum_{i=1}^{N}\frac{1}{n_{i}}\sum_{j=1}^{K}\sum_{k=1}^{n_{ij}}\ell_{i}\left(x_{k}^{i,j}+b_{i}\right)\leq z_{0}\right\}\\ \; & =\left\{\left({\tilde{y}}_{1}-b_{1}{\tilde{1}}_{1},\cdots ,{\tilde{y}}_{N}-b_{N}{\tilde{1}}_{N}\right)\in R^{n_{1}}\times \cdots \times R^{n_{N}}:\sum_{i=1}^{N}\frac{1}{n_{i}}\sum_{j=1}^{K}\sum_{k=1}^{n_{ij}}\ell_{i}\left(y_{k}^{i,j}\right)\leq z_{0}\right\}\\ \; & =\left\{\left({\tilde{y}}_{1},\cdots ,{\tilde{y}}_{N}\right)\in R^{n_{1}}\times \cdots \times R^{n_{N}}:\sum_{i=1}^{N}\frac{1}{n_{i}}\sum_{j=1}^{K}\sum_{k=1}^{n_{ij}}\ell_{i}\left(y_{k}^{i,j}\right)\leq z_{0}\right\}-\left\{(b_{1}{\tilde{1}}_{1},\cdots ,b_{N}{\tilde{1}}_{N})\right\}\\ \; & =B-\left\{(b_{1}{\tilde{1}}_{1},\cdots ,b_{N}{\tilde{1}}_{N})\right\}. \end{array}$$

From Proposition 1, we know $\tilde{B}=A_{\rho_{\tilde{B}}}$. Therefore, for any $\tilde{\omega }=\left({\tilde{\omega }}_{1},\cdots ,{\tilde{\omega }}_{N}\right)\in W$, we have that

(11) $$\begin{array}{cc} \underset{\left({\tilde{x}}_{1},\cdots ,{\tilde{x}}_{N}\right)\in A_{\rho_{\tilde{B}}}}{\sup}\sum_{i=1}^{N}\langle {\tilde{x}}_{i},{\tilde{\omega }}_{i}\rangle \; & =\underset{\left({\tilde{x}}_{1},\cdots ,{\tilde{x}}_{N}\right)\in \tilde{B}}{\sup}\sum_{i=1}^{N}\langle {\tilde{x}}_{i},{\tilde{\omega }}_{i}\rangle \\ \; & =\underset{({\tilde{y}}_{1},\cdots ,{\tilde{y}}_{N})\in B}{\sup}\sum_{i=1}^{N}\langle {\tilde{y}}_{i}-b_{i}{\tilde{1}}_{i},{\tilde{\omega }}_{i}\rangle \\ \; & =\underset{({\tilde{y}}_{1},\cdots ,{\tilde{y}}_{N})\in B}{\sup}\sum_{i=1}^{N}\langle {\tilde{y}}_{i},{\tilde{\omega }}_{i}\rangle -\sum_{i=1}^{N}b_{i}. \end{array}$$

Thus, if the assertion is established for $\tilde{L}$, then we find that

(12) $$\begin{array}{cc} {\tilde{\alpha }}_{min}\left(\tilde{\omega }\right)\; & =\underset{\lambda >0}{\inf}\frac{1}{\lambda }\left\{z_{0}+\sum_{i=1}^{N}\frac{1}{n_{i}}\sum_{j=1}^{K}\sum_{k=1}^{n_{ij}}{\tilde{\ell }}_{i}^{*}\left(\lambda n_{i}\omega_{k}^{i,j}\right)\right\}\\ \; & =\underset{\lambda >0}{\inf}\frac{1}{\lambda }\left\{z_{0}+\sum_{i=1}^{N}\frac{1}{n_{i}}\sum_{j=1}^{K}\sum_{k=1}^{n_{ij}}\left[\ell_{i}^{*}\left(\lambda n_{i}\omega_{k}^{i,j}\right)-b_{i}\lambda n_{i}\omega_{k}^{i,j}\right]\right\}\\ \; & =\underset{\lambda >0}{\inf}\frac{1}{\lambda }\left\{z_{0}+\sum_{i=1}^{N}\frac{1}{n_{i}}\sum_{j=1}^{K}\sum_{k=1}^{n_{ij}}\ell_{i}^{*}\left(\lambda n_{i}\omega_{k}^{i,j}\right)\right\}-\sum_{i=1}^{N}b_{i}, \end{array}$$

for any $\tilde{\omega }=\left({\tilde{\omega }}_{1},\cdots ,{\tilde{\omega }}_{N}\right)\in W$, where the second equation is implied by the fact that the Fenchel–Legendre transform ${\tilde{\ell }}_{i}^{*}$ of $\ell_{i}^{*}$ satisfies ${\tilde{\ell }}_{i}^{*}\left(x\right)=\ell_{i}^{*}\left(x\right)-b_{i}x$.

Note that $\rho_{\tilde{B}}$ is a multivariate convex risk statistic; by Lemma 1, we have that ${\tilde{\alpha }}_{min}\left(\tilde{\omega }\right)=\sup_{\left({\tilde{x}}_{1},\cdots ,{\tilde{x}}_{N}\right)\in A_{\rho_{\tilde{B}}}}\sum_{i=1}^{N}\langle {\tilde{x}}_{i},{\tilde{\omega }}_{i}\rangle$, for any $\tilde{\omega }=\left({\tilde{\omega }}_{1},\cdots ,{\tilde{\omega }}_{N}\right)\in W$, which, together with Equations (11) and (12), yields that

$$\begin{array}{c} \underset{({\tilde{y}}_{1},\cdots ,{\tilde{y}}_{N})\in B}{\sup}\sum_{i=1}^{N}\langle {\tilde{y}}_{i},{\tilde{\omega }}_{i}\rangle =\underset{\lambda >0}{\inf}\frac{1}{\lambda }\left\{z_{0}+\sum_{i=1}^{N}\frac{1}{n_{i}}\sum_{j=1}^{K}\sum_{k=1}^{n_{ij}}\ell_{i}^{*}\left(\lambda n_{i}\omega_{k}^{i,j}\right)\right\},\;\tilde{\omega }\in W. \end{array}$$

Therefore, it suffices to prove the claim for $L\left(\tilde{0}\right)<z_{0}$.

Next, we show the main proof process. Let us fix $\tilde{\omega }=\left({\tilde{\omega }}_{1},\cdots ,{\tilde{\omega }}_{N}\right)\in W$.

The part of “≤” in Equation (6). Note that $\ell_{i}^{*}$ is the conjugate function of $\ell_{i}$, $\ell_{i}^{*}\left(\omega_{k}^{i,j}\right)=\sup_{y\in R}\left\{\omega_{k}^{i,j}y-\ell_{i}\left(y\right)\right\}$. For any λ>0 and $\tilde{x}\in R^{n}$, we have that

$$\begin{array}{cc} x_{k}^{i,j}\omega_{k}^{i,j} & =\frac{1}{\lambda n_{i}}x_{k}^{i,j}\lambda n_{i}\omega_{k}^{i,j}\\ \; & \leq \frac{1}{\lambda n_{i}}\left[\ell_{i}\left(x_{k}^{i,j}\right)+\ell_{i}^{*}\left(\lambda n_{i}\omega_{k}^{i,j}\right)\right],i=1,\cdots ,N,j=1,\cdots ,K,k=1,\cdots ,n_{ij}, \end{array}$$

which yields that

$$\begin{array}{c} \sum_{i=1}^{N}\langle {\tilde{x}}_{i},{\tilde{\omega }}_{i}\rangle =\sum_{i=1}^{N}\sum_{j=1}^{k}\sum_{t=1}^{n_{ij}}x_{k}^{i,j}\omega_{k}^{i,j}\leq \frac{1}{\lambda }\left[\sum_{i=1}^{N}\frac{1}{n_{i}}\sum_{j=1}^{K}\sum_{k=1}^{n_{ij}}\ell_{i}\left(x_{k}^{i,j}\right)+\sum_{i=1}^{N}\frac{1}{n_{i}}\sum_{j=1}^{K}\sum_{k=1}^{n_{ij}}\ell_{i}^{*}\left(\lambda n_{i}\omega_{k}^{i,j}\right)\right]. \end{array}$$

Therefore,

$$\begin{array}{cc} \alpha_{min}\left(\tilde{\omega }\right) & =\underset{\left({\tilde{x}}_{1},\cdots ,{\tilde{x}}_{N}\right)\in A_{\rho_{B}}}{\sup}\sum_{i=1}^{N}\langle {\tilde{x}}_{i},{\tilde{\omega }}_{i}\rangle \\ \; & =\underset{({\tilde{x}}_{1},\cdots ,{\tilde{x}}_{N})\in B}{\sup}\sum_{i=1}^{N}\langle {\tilde{x}}_{i},{\tilde{\omega }}_{i}\rangle \\ \; & \leq \underset{({\tilde{x}}_{1},\cdots ,{\tilde{x}}_{N})\in B}{\sup}\frac{1}{\lambda }\left[\sum_{i=1}^{N}\frac{1}{n_{i}}\sum_{j=1}^{K}\sum_{k=1}^{n_{ij}}\ell_{i}\left(x_{k}^{i,j}\right)+\sum_{i=1}^{N}\frac{1}{n_{i}}\sum_{j=1}^{K}\sum_{k=1}^{n_{ij}}\ell_{i}^{*}\left(\lambda n_{i}\omega_{k}^{i,j}\right)\right]\\ \; & \leq \frac{1}{\lambda }\left[z_{0}+\sum_{i=1}^{N}\frac{1}{n_{i}}\sum_{j=1}^{K}\sum_{k=1}^{n_{ij}}\ell_{i}^{*}\left(\lambda n_{i}\omega_{k}^{i,j}\right)\right]. \end{array}$$

The part of “≥” in Equation (6). We will show that

(13) $$\begin{array}{c} \alpha_{min}\left(\tilde{\omega }\right)\geq \underset{\lambda >0}{\inf}\frac{1}{\lambda }\left[z_{0}+\sum_{i=1}^{N}\frac{1}{n_{i}}\sum_{j=1}^{K}\sum_{k=1}^{n_{ij}}\ell_{i}^{*}\left(\lambda n_{i}\omega_{k}^{i,j}\right)\right]. \end{array}$$

Without loss of generality, we assume that $\alpha_{min}\left(\tilde{\omega }\right)<\infty$. The proof of Equation (13) will be completed by three steps. First, the inequality Equation (13) will be proved under three extra conditions. Second, without the continuity condition, the result will be done. Finally, under the general case, we show the inequality.

Step 1. Assume that the following three conditions hold.

(C1) There exists $D\subseteq R^{n}$ such that $L\left(\tilde{x}\right)=\inf_{\tilde{y}\in R^{n}}L\left(\tilde{y}\right)$ for all $\tilde{x}\in D$, where *D* satisfies the monotonicity, i.e., if $\tilde{x}\in D$ then for any $\tilde{y}\leq \tilde{x}$, $\tilde{y}\in D$.
(C2) $\ell_{i},i=1,\cdots ,N$ are finite on (0,∞).
(C3) $J_{i},i=1,\cdots ,N$ are continuous on (0,∞).

Note that these assumptions imply that $\ell_{i}\left(0\right)<\infty$ and that $J_{i}\left(0+\right)$ have lower bounds. Moreover, $J_{i}\left(z\right)$ increases to *∞* as z↑∞, and hence so does $\ell_{i}\left(J_{i}\left(z\right)\right)$. Note that for all z∈R, $\ell_{i}^{*}\left(z\right)\geq -\ell_{i}\left(0\right),i=1,\cdots ,N,$ which yields that

(14) $$\begin{array}{c} \sum_{i=1}^{N}\frac{1}{n_{i}}\sum_{j=1}^{K}\sum_{k=1}^{n_{ij}}\ell_{i}^{*}\left(z_{k}^{i,j}\right)\geq \sum_{i=1}^{N}\frac{1}{n_{i}}\sum_{j=1}^{K}\sum_{k=1}^{n_{ij}}-\ell_{i}\left(0\right)=-L\left(\tilde{0}\right)>-z_{0}. \end{array}$$

From Equation (3), it follows that

$$\begin{array}{cc} \lim_{\tilde{z}↓\tilde{0}}\sum_{i=1}^{N}\frac{1}{n_{i}}\sum_{j=1}^{K}\sum_{k=1}^{n_{ij}}\ell_{i}\left(J_{i}\left(z_{k}^{i,j}\right)\right)-z_{0}\; & <\lim_{\tilde{z}↓\tilde{0}}\left[\sum_{i=1}^{N}\frac{1}{n_{i}}\sum_{j=1}^{K}\sum_{k=1}^{n_{ij}}\ell_{i}\left(J_{i}\left(z_{k}^{i,j}\right)\right)-\sum_{i=1}^{N}\frac{1}{n_{i}}\sum_{j=1}^{K}\sum_{k=1}^{n_{ij}}\ell_{i}^{*}\left(z_{k}^{i,j}\right)\right]\\ \; & =\lim_{\tilde{z}↓\tilde{0}}\sum_{i=1}^{N}\frac{1}{n_{i}}\sum_{j=1}^{K}\sum_{k=1}^{n_{ij}}z_{k}^{i,j}J_{i}\left(z_{k}^{i,j}\right)\\ \; & =0. \end{array}$$

These facts and the continuity of $J_{i}$ imply that, for large enough *m*, there exists some $\lambda_{m}>0$ such that

$$\begin{array}{c} \sum_{i=1}^{N}\frac{1}{n_{i}}\sum_{j=1}^{K}\sum_{k=1}^{n_{ij}}\ell_{i}\left(J_{i}\left(\lambda_{m}n_{i}\omega_{k}^{i,j}\right)I_{\left\{n_{i}\omega_{k}^{i,j}\leq m\right\}}\right)=z_{0}. \end{array}$$

Denote $x_{k}^{i,j}\left(m\right):=J_{i}\left(\lambda_{m}n_{i}\omega_{k}^{i,j}\right)I_{\left\{n_{i}\omega_{k}^{i,j}\leq m\right\}}$, $i=1,\cdots ,N,j=1,\cdots ,K,k=1,\cdots ,n_{ij}$, ${\tilde{x}}^{i,j}\left(m\right):=\left(x_{1}^{i,j}\left(m\right),\cdots ,x_{n_{ij}}^{i,j}\left(m\right)\right)$, ${\tilde{x}}^{i}\left(m\right):=\left({\tilde{x}}^{i,1}\left(m\right),\cdots ,{\tilde{x}}^{i,K}\left(m\right)\right)$ and $\tilde{x}\left(m\right)$:=$({\tilde{x}}^{1}\left(m\right),$⋯,${\tilde{x}}^{N}\left(m\right))$. Then $\tilde{x}\left(m\right)$ is bounded and belongs to B. Therefore, it follows from Equations (3) and (14) that

$$\begin{array}{cc} \alpha_{min}\left(\tilde{\omega }\right)\; & =\underset{({\tilde{x}}_{1},\cdots ,{\tilde{x}}_{N})\in B}{\sup}\sum_{i=1}^{N}\langle {\tilde{x}}_{i},{\tilde{\omega }}_{i}\rangle \\ \; & \geq \sum_{i=1}^{N}\langle {\tilde{x}}_{i}\left(m\right),{\tilde{\omega }}_{i}\rangle \\ \; & =\sum_{i=1}^{N}\sum_{j=1}^{K}\sum_{k=1}^{n_{ij}}J_{i}\left(\lambda_{m}n_{i}\omega_{k}^{i,j}\right)I_{\left\{n_{i}\omega_{k}^{i,j}\leq m\right\}}\omega_{k}^{i,j}\\ \; & =\frac{1}{\lambda_{m}}\sum_{i=1}^{N}\frac{1}{n_{i}}\sum_{j=1}^{K}\sum_{k=1}^{n_{ij}}I_{\left\{n_{i}\omega_{k}^{i,j}\leq m\right\}}J_{i}\left(\lambda_{m}n_{i}\omega_{k}^{i,j}\right)\lambda_{m}n_{i}\omega_{k}^{i,j}\\ \; & =\frac{1}{\lambda_{m}}\sum_{i=1}^{N}\frac{1}{n_{i}}\sum_{j=1}^{K}\sum_{k=1}^{n_{ij}}I_{\left\{n_{i}\omega_{k}^{i,j}\leq m\right\}}\left[\ell_{i}\left(J_{i}\left(\lambda_{m}n_{i}\omega_{k}^{i,j}\right)\right)+\ell_{i}^{*}\left(\lambda_{m}n_{i}\omega_{k}^{i,j}\right)\right]\\ \; & =\frac{1}{\lambda_{m}}\sum_{i=1}^{N}\frac{1}{n_{i}}\sum_{j=1}^{K}\sum_{k=1}^{n_{ij}}\left[\ell_{i}\left(J_{i}\left(\lambda_{m}n_{i}\omega_{k}^{i,j}\right)I_{\left\{n_{i}\omega_{k}^{i,j}\leq m\right\}}\right)-\ell_{i}\left(0\right)I_{\left\{n_{i}\omega_{k}^{i,j}>m\right\}}+\ell_{i}^{*}\left(\lambda_{m}n_{i}\omega_{k}^{i,j}\right)I_{\left\{n_{i}\omega_{k}^{i,j}\leq m\right\}}\right]\\ \; & =\frac{1}{\lambda_{m}}\left(z_{0}+\sum_{i=1}^{N}\frac{1}{n_{i}}\sum_{j=1}^{K}\sum_{k=1}^{n_{ij}}\left[-\ell_{i}\left(0\right)I_{\left\{n_{i}\omega_{k}^{i,j}>m\right\}}+\ell_{i}^{*}\left(\lambda_{m}n_{i}\omega_{k}^{i,j}\right)I_{\left\{n_{i}\omega_{k}^{i,j}\leq m\right\}}\right]\right)\\ \; & \geq \frac{1}{\lambda_{m}}\left(z_{0}-L\left(\tilde{0}\right)\right). \end{array}$$

As we assume that $\alpha_{min}\left(\tilde{\omega }\right)<\infty$, the limit $\lambda_{\infty }$ of $\left\{\lambda_{m}\right\}$ must be strictly positive.

$$\begin{array}{cc} \alpha_{min}\left(\tilde{\omega }\right) & \geq \lim_{m↑\infty }\frac{1}{\lambda_{m}}\left(z_{0}+\sum_{i=1}^{N}\frac{1}{n_{i}}\sum_{j=1}^{K}\sum_{k=1}^{n_{ij}}\left[-\ell_{i}\left(0\right)I_{\left\{n_{i}\omega_{k}^{i,j}>m\right\}}+\ell_{i}^{*}\left(\lambda_{m}n_{i}\omega_{k}^{i,j}\right)I_{\left\{n_{i}\omega_{k}^{i,j}\leq m\right\}}\right]\right)\\ \; & \geq \frac{1}{\lambda_{\infty }}\left(z_{0}+\sum_{i=1}^{N}\frac{1}{n_{i}}\sum_{j=1}^{K}\sum_{k=1}^{n_{ij}}\ell_{i}^{*}\left(\lambda_{\infty }n_{i}\omega_{k}^{i,j}\right)\right), \end{array}$$

which implies Equation (13).

Step 2. Assume that the conditions C1 and C2 hold, but not all $J_{i},i=1,\cdots ,N$ are continuous. Without loss of generality, we may assume $J_{i_{0}},i_{0}\in I_{0}$ are not continuous and the other $J_{i}$ are continuous. Then, we can approximate the function $J_{i_{0}}$ from above with an increasing continuous function ${\tilde{J}}_{i_{0}}$ on [0,∞) such that

$$\begin{array}{c} {\tilde{\ell }}_{i_{0}}^{*}\left(z\right):=\ell_{i_{0}}^{*}\left(0\right)+\int_{0}^{z}{\tilde{J}}_{i_{0}}\left(y\right)dy \end{array}$$

satisfies

$$\begin{array}{c} \ell_{i_{0}}^{*}\left(z\right)\leq {\tilde{\ell }}_{i_{0}}^{*}\left(z\right)\leq \ell_{i_{0}}^{*}\left(\left(1+\varepsilon \right)z\right),\;z\geq 0,\varepsilon >0. \end{array}$$

Denote ${\hat{\ell }}_{i_{0}}:={\tilde{\ell }}_{i_{0}}^{**}$, the Fenchel–Legendre transform of ${\tilde{\ell }}_{i_{0}}^{*}$. We can renew the loss functions such that the derivative $J_{i}$ is continuous for any i=1,⋯,N. As $\ell_{i}$ is a proper convex function, then $\ell_{i}^{**}=\ell_{i}$ and

$$\begin{array}{c} \ell_{i_{0}}\left(\frac{x}{1+\varepsilon }\right)\leq {\hat{\ell }}_{i_{0}}\left(x\right)\leq \ell_{i_{0}}\left(x\right). \end{array}$$

Thus,

$$\begin{array}{cc} \hat{B}: & =\left\{\tilde{x}\in R^{n}:\sum_{i\notin I_{0}}\frac{1}{n_{i}}\sum_{j=1}^{K}\sum_{k=1}^{n_{ij}}\ell_{i}\left(x_{k}^{i,j}\right)+\sum_{i\in I_{0}}\frac{1}{n_{i}}\sum_{j=1}^{K}\sum_{k=1}^{n_{ij}}{\hat{\ell }}_{i}\left(x_{k}^{i,j}\right)\leq z_{0}\right\}\\ \; & \subseteq \left\{\tilde{x}\in R^{n}:\sum_{i=1}^{N}\frac{1}{n_{i}}\sum_{j=1}^{K}\sum_{k=1}^{n_{ij}}\ell_{i}\left(\frac{x_{k}^{i,j}}{1+\varepsilon }\right)\leq z_{0}\right\}\\ \; & =(1+\varepsilon )B. \end{array}$$

By Step 1, we know that the assertion holds if the derivative $J_{i},i=1,\cdots ,N$ are continuous, which implies that

$$\begin{array}{cc} \; & \underset{\lambda >0}{\inf}\frac{1}{\lambda }\left(z_{0}+\sum_{i=1}^{N}\frac{1}{n_{i}}\sum_{j=1}^{K}\sum_{k=1}^{n_{ij}}\ell_{i}^{*}\left(\lambda n_{i}\omega_{k}^{i,j}\right)\right)\\ \; & \leq \underset{\lambda >0}{\inf}\frac{1}{\lambda }\left(z_{0}+\sum_{i\notin I_{0}}\frac{1}{n_{i}}\sum_{j=1}^{K}\sum_{k=1}^{n_{ij}}\ell_{i}^{*}\left(\lambda n_{i}\omega_{k}^{i,j}\right)+\sum_{i\in I_{0}}\frac{1}{n_{i}}\sum_{j=1}^{K}\sum_{k=1}^{n_{ij}}{\hat{\ell }}_{i}^{*}\left(\lambda n_{i}\omega_{k}^{i,j}\right)\right)\\ \; & =\underset{\left({\tilde{x}}_{1},\cdots ,{\tilde{x}}_{N}\right)\in \hat{B}}{\sup}\sum_{i=1}^{N}\langle {\tilde{x}}_{i},{\tilde{\omega }}_{i}\rangle \\ \; & \leq \underset{\left({\tilde{x}}_{1},\cdots ,{\tilde{x}}_{N}\right)\in \left(1+\varepsilon \right)B}{\sup}\sum_{i=1}^{N}\langle {\tilde{x}}_{i},{\tilde{\omega }}_{i}\rangle \\ \; & =\left(1+\varepsilon \right)\alpha_{min}\left(\tilde{\omega }\right). \end{array}$$

By letting ε↓0, we obtain inequality Equation (13).

Step 3. We remove conditions C1 and C2. Without loss of generality, suppose that there exists an $i_{0}$ such that $\ell_{i_{0}}^{*}\left(z\right)=+\infty$ for some *z*. Then, *z* must be an upper bound for the slope of $\ell_{i_{0}}$. Therefore, we approximate $\ell_{i_{0}}$ by a sequence ${\left\{\ell_{i_{0}}^{n}\right\}}_{n\in N}$ of convex loss functions whose slope is unbound. Simultaneously, we can handle the case where $\ell_{i_{0}}$ dose not take on its infimum. For this reason, we choose a sequence $z_{n}↓\inf_{x\in R}\ell_{i_{0}}$ with $z_{n}\leq \ell_{i_{0}}\left(0\right)<z_{0,i_{0}}$. Define

$$\begin{array}{c} \ell_{i_{0}}^{n}\left(x\right):=\max\left\{\ell_{i_{0}}\left(x\right),z_{n}\right\}+\frac{1}{n}{\left(e^{x}-1\right)}^{+}. \end{array}$$

Then, $\ell_{i_{0}}^{n}$ decreases to $\ell_{i_{0}}$, and each loss function $\ell_{i_{0}}^{n}$ satisfies the conditions C1 and C2. Therefore, for any n∈R and ε>0, there are $\lambda_{\varepsilon }^{n}$ such that

$$\begin{array}{c} \infty >\alpha_{min}\left(\tilde{\omega }\right)\geq \alpha_{min}^{n}\left(\tilde{\omega }\right)\geq \frac{1}{\lambda_{\varepsilon }^{n}}\left(z_{0}+{\left(\ell_{i_{0}}^{n}\right)}^{*}\left(\lambda_{\varepsilon }^{n}n_{i}\omega_{k}^{i,j}\right)+\sum_{i\neq i_{0}}\frac{1}{n_{i}}\sum_{j=1}^{K}\sum_{k=1}^{n_{ij}}\ell_{i}^{*}\left(\lambda_{\varepsilon }^{n}n_{i}\omega_{k}^{i,j}\right)\right)-\varepsilon , \end{array}$$

where $\alpha_{min}^{n}\left(\tilde{\omega }\right)$ is the penalty function arising from $\ell^{n}:=\left(\ell_{1},\cdots ,\ell_{i_{0}}^{n},\cdots ,\ell_{N}\right)$. Note that ${\left(\ell_{i_{0}}^{n}\right)}^{*}↗\ell_{i_{0}}^{*}$, by the assumption $\alpha_{min}\left(\tilde{\omega }\right)<\infty$, we have

(15) $$\begin{array}{cc} \; & \underset{z\in R}{\inf}{\left(\ell_{i_{0}}^{n}\right)}^{*}\left(z\right)\geq -\ell_{i_{0}}^{n}\left(0\right)=-\ell_{i_{0}}\left(0\right),\\ \; & \underset{\tilde{z}\in R^{n}}{\inf}\left\{{\left(\ell_{i_{0}}^{n}\right)}^{*}\left(z_{k}^{i,j}\right)+\sum_{i\neq i_{0}}\frac{1}{n_{i}}\sum_{j=1}^{K}\sum_{k=1}^{n_{ij}}\ell_{i}^{*}\left(z_{k}^{i,j}\right)\right\}\geq -L\left(\tilde{0}\right)>-z_{0}. \end{array}$$

As $\frac{{\left(\ell_{i}^{n}\right)}^{*}\left(z\right)}{z}\to \infty$ as z↑∞, the sequence ${\left\{\lambda_{\varepsilon }^{n}\right\}}_{n\in N}$ must be bounded away from zero and from infinity. Therefore, we assume that $\lambda_{\varepsilon }^{n}$ converges to some $\lambda_{\varepsilon }\in \left(0,\infty \right)$. Using again the fact that Equation (15) uniformly in *n* and *z*, we have that

$$\begin{array}{cc} \alpha_{min}\left(\tilde{\omega }\right)+\varepsilon \; & \geq \underset{n↑\infty }{lim inf}\frac{1}{\lambda_{\varepsilon }^{n}}\left(z_{0}+{\left(\ell_{i_{0}}^{n}\right)}^{*}\left(\lambda_{\varepsilon }^{n}n_{i}\omega_{k}^{i,j}\right)+\sum_{i\neq i_{0}}\frac{1}{n_{i}}\sum_{j=1}^{K}\sum_{k=1}^{n_{ij}}\ell_{i}^{*}\left(\lambda_{\varepsilon }^{n}n_{i}\omega_{k}^{i,j}\right)\right)\\ \; & \geq \frac{1}{\lambda_{\varepsilon }}\left(z_{0}+\sum_{i=1}^{N}\frac{1}{n_{i}}\sum_{j=1}^{K}\sum_{k=1}^{n_{ij}}\ell_{i}^{*}\left(\lambda_{\varepsilon }n_{i}\omega_{k}^{i,j}\right)\right). \end{array}$$

The proof of Theorem 1 is completed. □

**Proof** **of** **Corollary** **1.** We will first show that

(16) $$\begin{array}{c} \rho_{L}\left(\tilde{x}\right)=\underset{\ell \in L}{\sup}\rho_{\ell }\left(\tilde{x}\right), \end{array}$$

for any $\tilde{x}=\left(x_{1,1},\cdots ,x_{1,n_{1}},x_{2,1},\cdots ,x_{K,n_{K}}\right)\in R^{n}$, where for ℓ∈L,

$$\begin{array}{c} \rho_{\ell }\left(\tilde{x}\right):=\rho_{A_{\left(\ell ,z_{0}\right)}}\left(\tilde{x}\right):=\inf\left\{m\in R:\frac{1}{n}\sum_{j=1}^{K}\sum_{k=1}^{n_{j}}\ell \left(x_{jk}-m\right)\leq z_{0}\right\}. \end{array}$$

Note that for any $\tilde{x}\in R^{n}$,

$$\begin{array}{c} \rho_{L}\left(\tilde{x}\right)=\inf\left\{m\in R:\frac{1}{n}\sum_{j=1}^{K}\sum_{k=1}^{n_{j}}\ell \left(x_{jk}-m\right)\leq z_{0}\;\mathrm{for}\;\mathrm{all}\;\ell \in L\right\}. \end{array}$$

Therefore, for any ℓ∈L and $\tilde{x}\in R^{n}$,

$$\begin{array}{c} \rho_{L}\left(\tilde{x}\right)\geq \inf\left\{m\in R:\frac{1}{n}\sum_{j=1}^{K}\sum_{k=1}^{n_{j}}\ell \left(x_{jk}-m\right)\leq z_{0}\right\}=\rho_{\ell }\left(\tilde{x}\right), \end{array}$$

which implies that

$$\begin{array}{c} \rho_{L}\left(\tilde{x}\right)\geq \underset{\ell \in L}{\sup}\rho_{\ell }\left(\tilde{x}\right). \end{array}$$

Next, we will show that

$$\begin{array}{c} \rho_{L}\left(\tilde{x}\right)\leq \underset{\ell \in L}{\sup}\rho_{\ell }\left(\tilde{x}\right). \end{array}$$

Without loss of generality, we assume that $M:=\sup_{\ell \in L}\rho_{\ell }\left(\tilde{x}\right)<+\infty$. For any ℓ∈L,

$$\begin{array}{c} M\geq \rho_{\ell }\left(\tilde{x}\right)=\inf\left\{m\in R:\frac{1}{n}\sum_{j=1}^{K}\sum_{k=1}^{n_{j}}\ell \left(x_{jk}-m\right)\leq z_{0}\right\}. \end{array}$$

Therefore, for every ε>0, there exists an $m_{\ell }=m\left(\ell ,\varepsilon \right)$ with $\frac{1}{n}\sum_{j=1}^{K}\sum_{k=1}^{n_{j}}\ell \left(x_{jk}-m_{\ell }\right)\leq z_{0}$ such that

$$\begin{array}{c} m_{\ell }\leq \rho_{\ell }\left(\tilde{x}\right)+\varepsilon . \end{array}$$

Thus, for any ℓ∈L,

$$\begin{array}{c} -\infty <m_{\ell }\leq \rho_{\ell }\left(\tilde{x}\right)+\varepsilon \leq M+\varepsilon <+\infty , \end{array}$$

which yields that

(17) $$\begin{array}{c} -\infty <\underset{\ell \in L}{\sup}m_{\ell }\leq M+\varepsilon .P \end{array}$$

For any ℓ∈L, by the increasing property of *ℓ*,

$$\begin{array}{c} \frac{1}{n}\sum_{j=1}^{K}\sum_{k=1}^{n_{j}}\ell \left(x_{jk}-\underset{\ell \in L}{\sup}m_{\ell }\right)\leq \frac{1}{n}\sum_{j=1}^{K}\sum_{k=1}^{n_{j}}\ell \left(x_{jk}-m_{\ell }\right)\leq z_{0}, \end{array}$$

which yields that

$$\begin{array}{c} \underset{\ell \in L}{\sup}m_{\ell }\in \left\{m\in R:\frac{1}{n}\sum_{j=1}^{K}\sum_{k=1}^{n_{j}}\ell \left(x_{jk}-m\right)\leq z_{0}\;\mathrm{for}\;\mathrm{all}\;\ell \in L\right\}. \end{array}$$

By the definition of $\rho_{L}$ and Equation (17), we know that

$$\begin{array}{c} \rho_{L}\left(\tilde{x}\right)\leq \underset{\ell \in L}{\sup}m_{\ell }\leq M+\varepsilon , \end{array}$$

which implies that

$$\begin{array}{c} \rho_{L}\left(\tilde{x}\right)\leq \underset{\ell \in L}{\sup}\rho_{\ell }\left(\tilde{x}\right), \end{array}$$

since ε>0 is arbitrary.

From Theorem 1 it follows that for any ℓ∈L,

(18) $$\begin{array}{c} \rho_{\ell }\left(\tilde{x}\right)=\underset{\tilde{\omega }\in \tilde{W}}{\sup}\left\{\langle \tilde{x},\tilde{\omega }\rangle -\alpha_{\min}^{\ell }\left(\tilde{w}\right)\right\}, \end{array}$$

for $\tilde{x}\in R^{n}$, where the minimal penalty function $\alpha_{\min}^{\ell }:R^{n}\to R$ is given by

$$\begin{array}{c} \alpha_{\min}^{\ell }\left(\tilde{\omega }\right)=\underset{\lambda >0}{\inf}\left\{\frac{z_{0}\left(\ell \right)}{\lambda }+\frac{1}{\lambda n}\sum_{j=1}^{K}\sum_{k=1}^{n_{j}}\ell^{*}\left(\lambda n\omega_{jk}\right)\right\}. \end{array}$$

Therefore, from Equations (16) and (18), it follows that

$$\begin{array}{cc} \rho_{L}\left(\tilde{x}\right)\; & =\underset{\ell \in L}{\sup}\rho_{\ell }\left(\tilde{x}\right)\\ \; & =\underset{\ell \in L}{\sup}\underset{\tilde{\omega }\in \tilde{W}}{\sup}\left\{\langle \tilde{x},\tilde{\omega }\rangle -\alpha_{\min}^{\ell }\left(\tilde{w}\right)\right\}\\ \; & =\underset{\tilde{\omega }\in \tilde{W}}{\sup}\left\{\langle \tilde{x},\tilde{\omega }\rangle -\underset{\ell \in L}{\inf}\alpha_{\min}^{\ell }\left(\tilde{w}\right)\right\}. \end{array}$$

Therefore, the minimal penalty function $\alpha_{\min}:R^{n}\to R$ is

$$\begin{array}{cc} \alpha_{\min}\left(\tilde{w}\right)\; & =\underset{\ell \in L}{\inf}\alpha_{\min}^{\ell }\left(\tilde{w}\right)\\ \; & =\underset{\ell \in L}{\inf}\underset{\lambda >0}{\inf}\left\{\frac{z_{0}\left(\ell \right)}{\lambda }+\frac{1}{\lambda n}\sum_{j=1}^{K}\sum_{k=1}^{n_{j}}\ell^{*}\left(\lambda n\omega_{jk}\right)\right\}\\ \; & =\underset{\lambda >0}{\inf}\underset{\ell \in L}{\inf}\left\{\frac{z_{0}\left(\ell \right)}{\lambda }+\frac{1}{\lambda n}\sum_{j=1}^{K}\sum_{k=1}^{n_{j}}\ell^{*}\left(\lambda n\omega_{jk}\right)\right\}, \end{array}$$

for $\tilde{\omega }\in \tilde{W}$. The proof of Corollary 1 is completed. □

**Proof** **of** **Theorem** **2.** Sufficiency: Suppose that $\ell \left(x\right)=z_{0}+\alpha x^{+}-\beta x^{-}$ with some 0<β≤α. Since 0<β≤α, the shortfall risk statistic $\rho_{A}$ is a convex risk statistic by Proposition 3.

For any $\tilde{x}=\left(x_{1,1},\cdots ,x_{1,n_{1}},x_{2,1},\cdots ,x_{K,n_{K}}\right)\in R^{n}$ and λ>0,

$$\begin{array}{cc} \rho_{B}\left(\lambda \tilde{x}\right)\; & =\inf\left\{m\in R:\frac{1}{n}\sum_{j=1}^{K}\sum_{k=1}^{n_{j}}\ell \left(\lambda x_{jk}-m\right)\leq z_{0}\right\}\\ \; & =\inf\left\{m\in R:\frac{1}{n}\sum_{j=1}^{K}\sum_{k=1}^{n_{j}}\left[\frac{z_{0}}{n}+\alpha {\left(\lambda x_{jk}-m\right)}^{+}-\beta {\left(\lambda x_{jk}-m\right)}^{-}\right]\leq z_{0}\right\}\\ \; & =\inf\left\{m\in R:\frac{1}{n}\sum_{j=1}^{K}\sum_{k=1}^{n_{j}}\left[\alpha {\left(\lambda x_{jk}-m\right)}^{+}-\beta {\left(\lambda x_{jk}-m\right)}^{-}\right]\leq 0\right\}\\ \; & =\inf\left\{m\in R:\frac{1}{n}\sum_{j=1}^{K}\sum_{k=1}^{n_{j}}\left[\alpha {\left(x_{jk}-\frac{m}{\lambda }\right)}^{+}-\beta {\left(x_{jk}-\frac{m}{\lambda }\right)}^{-}\right]\leq 0\right\}\\ \; & =\inf\left\{\lambda m^{*}\in R:\frac{1}{n}\sum_{j=1}^{K}\sum_{k=1}^{n_{j}}\left[\frac{z_{0}}{n}+\alpha {\left(x_{jk}-m^{*}\right)}^{+}-\beta {\left(x_{jk}-m^{*}\right)}^{-}\right]\leq z_{0}\right\}\\ \; & =\inf\left\{\lambda m^{*}\in R:\frac{1}{n}\sum_{j=1}^{K}\sum_{k=1}^{n_{j}}\ell \left(x_{jk}-m^{*}\right)\leq z_{0}\right\}\\ \; & =\lambda \rho_{B}\left(\tilde{x}\right), \end{array}$$

which implies that $\rho_{B}$ is positively homogenous, and hence $\rho_{B}$ is a coherent risk statistic.

Necessity: Suppose that $\rho_{B}$ is coherent. Denote $\tilde{\ell }\left(x\right):=\ell \left(x\right)-z_{0}$ and

$$\begin{array}{c} \hat{B}:=\left\{\tilde{x}=\left(x_{1,1},\cdots ,x_{1,n_{1}},x_{2,1},\cdots ,x_{K,n_{K}}\right)\in R^{n}:\frac{1}{n}\sum_{j=1}^{K}\sum_{k=1}^{n_{j}}\tilde{\ell }\left(x_{jk}\right)\leq 0\right\}. \end{array}$$

Then, $\hat{B}=\left\{\tilde{x}\in R^{n}:\frac{1}{n}\sum_{j=1}^{K}\sum_{k=1}^{n_{j}}\left[\ell \left(x_{jk}\right)-z_{0}\right]\leq 0\right\}=B$.

By the positive homogeneity and continuity of $\rho_{B}$, for any $\tilde{x}\in R^{n}$ and λ>0, $\rho_{B}\left(\lambda \tilde{x}\right)=\lambda \rho_{B}\left(\tilde{x}\right)$, thus $\rho_{B}\left(\tilde{0}\right)=0$, and therefore

$$\begin{array}{cc} 0=\rho_{B}\left(\tilde{0}\right) & =\inf\left\{m\in R:\frac{1}{n}\sum_{j=1}^{K}\sum_{k=1}^{n_{j}}\tilde{\ell }\left(0-m\right)\leq 0\right\}\\ \; & =\inf\left\{m\in R:\tilde{\ell }\left(-m\right)\leq 0\right\}. \end{array}$$

As the convex function $\tilde{\ell }$ is continuous and strictly increasing, we have $\tilde{\ell }\left(0\right)=0$.

Denote

$$\begin{array}{c} A_{\rho_{B}}:=\left\{\tilde{x}\in R^{n}:\rho_{B}\left(\tilde{x}\right)\leq 0\right\}. \end{array}$$

Then the positive homogeneity of $\rho_{A}$ implies that $A_{\rho_{B}}$ is a cone, i.e., $\lambda \tilde{x}\in A_{\rho_{A}}$ for any $\tilde{x}\in A_{\rho_{A}}$ and λ>0. By Proposition 1, $B=A_{\rho_{B}}$ and B is a cone.

Next, we will show that $\tilde{\ell }\left(\lambda x\right)=\lambda \tilde{\ell }\left(x\right)$ for any x∈R and λ>0. In fact, suppose that there exist $x_{0}\in R$ and $\lambda_{0}>0$ such that $\tilde{\ell }\left(\lambda_{0}x_{0}\right)\neq \lambda_{0}\tilde{\ell }\left(x_{0}\right)$. Without loss of generality, we assume that $\lambda_{0}>1$. Otherwise, if $0<\lambda_{0}<1$, then denote $\lambda_{0}^{{}^{\prime }}:=\frac{1}{\lambda_{0}},x_{0}^{{}^{\prime }}:=\lambda_{0}x_{0}$, and thus $\tilde{\ell }\left(\lambda_{0}^{{}^{\prime }}x_{0}^{{}^{\prime }}\right)\neq \lambda_{0}^{{}^{\prime }}\tilde{\ell }\left(x_{0}^{{}^{\prime }}\right)$.

By the convexity of $\tilde{\ell }$ and $\tilde{\ell }\left(0\right)=0$, we have that

(19) $$\begin{array}{cc} \frac{1}{\lambda_{0}}\tilde{\ell }\left(\lambda_{0}x_{0}\right)\; & =\left(1-\frac{1}{\lambda_{0}}\right)\tilde{\ell }\left(0\right)+\frac{1}{\lambda_{0}}\tilde{\ell }\left(\lambda_{0}x_{0}\right)\\ \; & \geq \tilde{\ell }\left[\left(1-\frac{1}{\lambda_{0}}\right)\cdot 0+\frac{1}{\lambda_{0}}\lambda_{0}x_{0}\right]\\ \; & =\tilde{\ell }\left(x_{0}\right), \end{array}$$

which yields that $\tilde{\ell }\left(\lambda_{0}x_{0}\right)>\lambda_{0}\tilde{\ell }\left(x_{0}\right)$.

Recall that the convex function *ℓ* is continuous with $\inf_{x\in R}\ell \left(x\right)=-\infty$ and $\sup_{x\in R}\ell \left(x\right)=+\infty$, by the intermediate value theorem for continuous function, there exist $\left(x_{2},\cdots ,x_{n}\right)\in R^{n-1}$ such that

$$\begin{array}{c} \tilde{\ell }\left(x_{0}\right)+\tilde{\ell }\left(x_{2}\right)+\cdots +\tilde{\ell }\left(x_{n}\right)=0. \end{array}$$

Therefore, by the definition of the acceptance set $\hat{B}$, $\left(x_{0},x_{2},\cdots ,x_{n}\right)\in \hat{B}=B$.

Similar to Equation (19), we know that $\tilde{\ell }\left(\lambda_{0}x\right)\geq \lambda_{0}\tilde{\ell }\left(x\right)$ for any x∈R, therefore

$$\begin{array}{cc} \tilde{\ell }\left(\lambda_{0}x_{0}\right)+\tilde{\ell }\left(\lambda_{0}x_{2}\right)+\cdots +\tilde{\ell }\left(\lambda_{0}x_{n}\right)\; & \geq \tilde{\ell }\left(\lambda_{0}x_{0}\right)+\lambda_{0}\tilde{\ell }\left(x_{2}\right)+\cdots +\lambda_{0}\tilde{\ell }\left(x_{n}\right)\\ \; & >\lambda_{0}\tilde{\ell }\left(x_{0}\right)+\lambda_{0}\tilde{\ell }\left(x_{2}\right)+\cdots +\lambda_{0}\tilde{\ell }\left(x_{n}\right)\\ \; & =\lambda_{0}\left[\tilde{\ell }\left(x_{0}\right)+\tilde{\ell }\left(x_{2}\right)+\cdots +\tilde{\ell }\left(x_{n}\right)\right]\\ \; & =0. \end{array}$$

Thus $\lambda_{0}\left(x_{0},x_{2},\cdots ,\lambda_{0}x_{n}\right)=\left(\lambda_{0}x_{0},\lambda_{0}x_{2},\cdots ,\lambda_{0}\lambda_{0}x_{n}\right)\notin B$, which contradicts the fact that B is a cone.

Therefore, $\lambda \tilde{\ell }\left(x\right)=\tilde{\ell }\left(\lambda x\right)$ for any x∈R and λ>0. This results in that

$$\tilde{\ell }\left(x\right)=\alpha x^{+}-\beta x^{-},x\in R$$

with some α>0,β>0, since $\tilde{\ell }$ is increasing. The inequality β≤α follows from the convexity of $\tilde{\ell }$. Consequently, $\ell \left(x\right)=z_{0}+\alpha x^{+}-\beta x^{-}$ for all x∈R with some 0<β≤α<+∞. The proof of Theorem 2 is completed. □

**Proof** **of** **Theorem** **3.** We will make full use of convex analysis to show theorem.

Define ${\tilde{g}}_{i},i=1,\cdots ,N$ and *G* as the same way as in Remark 2. It is easy to check that $\ell_{i}:=g_{i}^{*}$ satisfies the assumptions in Theorem 1 for each 1≤i≤N. As $\ell_{i}^{*}=g_{i}^{**}=g_{i},1\leq i\leq N$, Theorem 1 implies that for fixed $\tilde{x}\in R^{n_{1}}\times \cdots \times R^{n_{N}}$

$$\begin{array}{cc} F\left(s\right):=F_{\tilde{x}}\left(s\right): & =\inf\left\{\sum_{i=1}^{N}z_{i}:\sum_{i=1}^{N}\frac{1}{n_{i}}\sum_{j=1}^{K}\sum_{k=1}^{n_{ij}}g_{i}^{*}\left(x_{k}^{i,j}-z_{i}\right)\leq s\right\}\\ \; & =\underset{\tilde{\omega }\in W}{\sup}\left\{\langle \tilde{x},\tilde{\omega }\rangle -\underset{\lambda >0}{\inf}\frac{1}{\lambda }\left\{s+\sum_{i=1}^{N}\frac{1}{n_{i}}\sum_{j=1}^{K}\sum_{k=1}^{n_{ij}}g_{i}\left(\lambda n_{i}\omega_{k}^{i,j}\right)\right\}\right\}\\ \; & =-\underset{\tilde{\omega }\in W}{\inf}\left\{-\langle \tilde{x},\tilde{\omega }\rangle +\underset{\beta >0}{\inf}\left\{\beta s+\sum_{i=1}^{N}\frac{1}{n_{i}}\sum_{j=1}^{K}\sum_{k=1}^{n_{ij}}\beta g_{i}\left(\frac{n_{i}\omega_{k}^{i,j}}{\beta }\right)\right\}\right\}\\ \; & =-\underset{\beta >0}{\inf}\underset{\tilde{\omega }\in W}{\inf}\left\{-\langle \tilde{x},\tilde{\omega }\rangle +\beta s+\sum_{i=1}^{N}\frac{1}{n_{i}}\sum_{j=1}^{K}\sum_{k=1}^{n_{ij}}\beta g_{i}\left(\frac{n_{i}\omega_{k}^{i,j}}{\beta }\right)\right\}\\ \; & =-\underset{\beta >0}{\inf}\left\{\beta s+\underset{\tilde{\omega }\in W}{\inf}\left\{-\langle \tilde{x},\tilde{\omega }\rangle +\sum_{i=1}^{N}\frac{1}{n_{i}}\sum_{j=1}^{K}\sum_{k=1}^{n_{ij}}\beta g_{i}\left(\frac{n_{i}\omega_{k}^{i,j}}{\beta }\right)\right\}\right\}\\ \; & =-\underset{\beta >0}{\inf}\left\{\beta s+\underset{\tilde{\omega }\in W}{\inf}\left\{-\langle \tilde{x},\tilde{\omega }\rangle +I_{\left({\tilde{g}}_{1},\cdots ,{\tilde{g}}_{N}\right)}\left(\beta ,\tilde{\omega }\right)\right\}\right\}\\ \; & =-\underset{\beta >0}{\inf}\left\{\beta s+G(\beta )\right\}\\ \; & =\underset{\beta >0}{\sup}\left\{\beta \dot{(}-s)-G\left(\beta \right)\right\}\\ \; & =G^{*}\left(-s\right), \end{array}$$

for all *s* in the interior of set $\left\{\sum_{i=1}^{N}\frac{1}{n_{i}}\sum_{j=1}^{K}\sum_{k=1}^{n_{ij}}g_{i}^{*}\left(x_{k}^{i,j}\right):\tilde{x}\in R^{n_{1}}\times \cdots \times R^{n_{N}}\right\}$, which coincides with the interior of dom*F*.

Note that for t∈R,

$$\begin{array}{c} G\left(t\right)=G^{**}\left(t\right)=\underset{-s\in \mathrm{dom}F}{\sup}\left\{st-G^{*}\left(s\right)\right\}=\underset{-s\in \mathrm{dom}F}{\sup}\left\{st-F\left(-s\right)\right\}. \end{array}$$

By the definition of *G*,

$$\begin{array}{c} \rho_{\left(g_{1},\cdots ,g_{N}\right)}\left(\tilde{x}\right)=-G\left(1\right)=-\underset{-s\in \mathrm{dom}F}{\sup}\left\{s-F\left(-s\right)\right\}=\underset{s\in \mathrm{dom}F}{\inf}\left\{s+F\left(s\right)\right\}. \end{array}$$

For any s∈domF, there exists ${\tilde{z}}^{*}:={\tilde{z}}^{*}\left(g_{1},\cdots ,g_{N},\tilde{x}\right):=\left(z_{1}^{*},\cdots ,z_{N}^{*}\right)\in R^{N}$ with

$$\begin{array}{c} \sum_{i=1}^{N}\frac{1}{n_{i}}\sum_{j=1}^{K}\sum_{k=1}^{n_{ij}}g_{i}^{*}\left(x_{k}^{i,j}-z_{i}^{*}\right)=s \end{array}$$

such that

$$\begin{array}{c} \sum_{i=1}^{N}z_{i}^{*}=F\left(s\right), \end{array}$$

as $g_{i}^{*}$ is continuous for each 1≤i≤N.

Thus, for any $\tilde{x}\in R^{n_{1}}\times \cdots \times R^{n_{N}}$, the multivariate divergence risk statistic can be rewritten as

$$\begin{array}{c} \rho_{\left(g_{1},\cdots ,g_{N}\right)}\left(\tilde{x}\right)=\underset{s\in \mathrm{dom}F}{\inf}\left\{s+F\left(s\right)\right\}=\underset{\left(z_{1},\cdots ,z_{N}\right)\in R^{N}}{\inf}\left\{\sum_{i=1}^{N}\frac{1}{n_{i}}\sum_{j=1}^{K}\sum_{k=1}^{n_{ij}}g_{i}^{*}\left(x_{k}^{i,j}-z_{i}\right)+\sum_{i=1}^{N}z_{i}\right\}. \end{array}$$

The proof of Theorem 3 is completed. □

## 5. Examples

In this section, we will construct entropic (or entropy-like) risk statistics by choosing specific loss functions.

**Example** **1.** *For exponential loss functions* $\ell_{i}\left(x\right):=\frac{e^{\beta_{i}x}-1}{\beta_{i}},\beta_{i}>0$ *, and* $z_{0,i}>-\frac{1}{\beta_{i}}$ *for each* 1≤i≤N *, then* $z_{0}+\sum_{i=1}^{N}\frac{1}{\beta_{i}}=\sum_{i=1}^{N}\left(z_{0,i}+\frac{1}{\beta_{i}}\right)>0$ *. we know that the conjugation function* $\ell_{i}^{*}:\left[0,+\infty \right)$ *is defined as*

$$\begin{array}{c} \ell_{i}^{*}\left(x\right)=\left\{\begin{array}{c} \frac{x}{\beta_{i}}\log x-\frac{x}{\beta_{i}}+\frac{1}{\beta_{i}},\;if\;x>0,\\ \frac{1}{\beta_{i}},\;\;\;\;\;\;\;x=0. \end{array}\right. \end{array}$$

*Then by the definition of the shortfall risk statistic in (5), we know that the corresponding shortfall risk statistic is*

(20) $$\begin{array}{cc} \rho_{B}\left(\tilde{x}\right) & =\inf\left\{\sum_{i=1}^{N}m_{i}:\sum_{i=1}^{N}\frac{1}{n_{i}}\sum_{j=1}^{K}\sum_{k=1}^{n_{ij}}\ell_{i}\left(x_{k}^{i,j}-m_{i}\right)\leq z_{0}\right\}\\ \; & =\inf\left\{\sum_{i=1}^{N}m_{i}:\sum_{i=1}^{N}\frac{1}{n_{i}\beta_{i}}\sum_{j=1}^{K}\sum_{k=1}^{n_{ij}}e^{\beta_{i}\left(x_{k}^{i,j}-m_{i}\right)}\leq z_{0}+\sum_{i=1}^{N}\frac{1}{\beta_{i}}\right\} \end{array}$$

*for* $\tilde{x}=\left({\tilde{x}}_{1},\cdots ,{\tilde{x}}_{N}\right)=\left(x_{1}^{1,1},\cdots ,x_{n_{11}}^{1,1},\cdots ,x_{1}^{1,K},\cdots ,x_{n_{1k}}^{1,K},\cdots ,x_{1}^{N,1},\cdots ,x_{n_{N1}}^{N,1},\cdots ,x_{1}^{N,K},\cdots ,x_{n_{NK}}^{N,K}\right)\in R^{n_{1}}\times \cdots \times R^{n_{N}}$ *.*

*By Theorem 1, we have that the penalty function of* $\rho_{B}$ *is*

(21) $$\begin{array}{cc} \alpha_{min}\left(\tilde{\omega }\right)\; & =\underset{\lambda >0}{\inf}\frac{1}{\lambda }\left\{z_{0}+\sum_{i=1}^{N}\frac{1}{n_{i}}\sum_{j=1}^{K}\sum_{k=1}^{n_{ij}}\ell_{i}^{*}\left(\lambda n_{i}\omega_{k}^{i,j}\right)\right\}\\ \; & =\underset{\lambda >0}{\inf}\frac{1}{\lambda }\left\{z_{0}+\sum_{i=1}^{N}\frac{1}{n_{i}}\sum_{j=1}^{K}\sum_{k=1}^{n_{ij}}\left[\frac{\lambda n_{i}\omega_{k}^{i,j}}{\beta_{i}}\log\lambda n_{i}\omega_{k}^{i,j}-\frac{\lambda n_{i}\omega_{k}^{i,j}}{\beta_{i}}+\frac{1}{\beta_{i}}\right]\right\}\\ \; & =\sum_{i=1}^{N}\frac{1}{\beta_{i}}\sum_{j=1}^{K}\sum_{k=1}^{n_{ij}}\omega_{k}^{i,j}\log\left[\frac{z_{0}+\sum_{i=1}^{N}\frac{1}{\beta_{i}}}{\sum_{i=1}^{N}\frac{1}{\beta_{i}}}n_{i}\omega_{k}^{i,j}\right] \end{array}$$

*for any* $\tilde{\omega }\in W=\left\{\tilde{\omega }=\left({\tilde{\omega }}_{1},\cdots ,{\tilde{\omega }}_{N}\right)\in R^{n_{1}}\times \cdots \times R^{n_{N}}:\tilde{\omega }\geq 0,\langle {\tilde{1}}_{i},{\tilde{\omega }}_{i}\rangle =1,i=1,\cdots ,N\right\}$ *, where the infimum is arrived at* $\lambda =\frac{z_{0}+\sum_{i=1}^{N}\frac{1}{\beta_{i}}}{\sum_{i=1}^{N}\frac{1}{\beta_{i}}}$ *.*

*Furthermore, by Theorem 1, we know that* $\rho_{B}$ *has the following dual representation,*

(22) $$\begin{array}{cc} \rho_{B}\left(\tilde{x}\right) & =\underset{\tilde{\omega }\in W}{\sup}\left\{\langle \tilde{x},\tilde{\omega }\rangle -\underset{\lambda >0}{\inf}\frac{1}{\lambda }\left\{z_{0}+\sum_{i=1}^{N}\frac{1}{n_{i}}\sum_{j=1}^{K}\sum_{k=1}^{n_{ij}}\ell_{i}^{*}\left(\lambda n_{i}\omega_{k}^{i,j}\right)\right\}\right\}\\ \; & =\underset{\tilde{\omega }\in W}{\sup}\left\{\langle \tilde{x},\tilde{\omega }\rangle -\sum_{i=1}^{N}\frac{1}{\beta_{i}}\sum_{j=1}^{K}\sum_{k=1}^{n_{ij}}\omega_{k}^{i,j}\log\left[\frac{z_{0}+\sum_{i=1}^{N}\frac{1}{\beta_{i}}}{\sum_{i=1}^{N}\frac{1}{\beta_{i}}}n_{i}\omega_{k}^{i,j}\right]\right\}. \end{array}$$

*Let* $g_{i}\left(x\right):=\ell_{i}^{*}\left(x\right)$ *for each* 1≤i≤N *, the divergence function* $I_{\left(g_{1},\cdots ,g_{N}\right)}:{\left[0,+\infty \right)}^{n_{1}}\times \cdots \times {\left[0,+\infty \right)}^{n_{N}}\to R$ *is defined by*

(23) $$\begin{array}{cc} I_{\left(g_{1},\cdots ,g_{N}\right)}\left(\tilde{\omega }\right) & =\sum_{i=1}^{N}\frac{1}{n_{i}}\sum_{j=1}^{K}\sum_{k=1}^{n_{ij}}g_{i}\left(n_{i}\omega_{k}^{i,j}\right)\\ \; & =\sum_{i=1}^{N}\frac{1}{n_{i}}\sum_{j=1}^{K}\sum_{k=1}^{n_{ij}}\left(\frac{n_{i}\omega_{k}^{i,j}}{\beta_{i}}\log\left(n_{i}\omega_{k}^{i,j}\right)-\frac{n_{i}\omega_{k}^{i,j}}{\beta_{i}}+\frac{1}{\beta_{i}}\right)\\ \; & =\sum_{i=1}^{N}\frac{1}{\beta_{i}}\sum_{j=1}^{K}\sum_{k=1}^{n_{ij}}\omega_{k}^{i,j}\log\left(n_{i}\omega_{k}^{i,j}\right). \end{array}$$

*Therefore, by Theorem 3, the divergence risk statistic is*

(24) $$\begin{array}{cc} \rho_{\left(g_{1},\cdots ,g_{N}\right)}\left(\tilde{x}\right) & =\underset{\left(z_{1},\cdots ,z_{N}\right)\in R^{N}}{\inf}\left\{\sum_{i=1}^{N}\frac{1}{n_{i}}\sum_{j=1}^{K}\sum_{k=1}^{n_{ij}}g_{i}^{*}\left(x_{k}^{i,j}-z_{i}\right)+\sum_{i=1}^{N}z_{i}\right\}\\ \; & =\underset{\left(z_{1},\cdots ,z_{N}\right)\in R^{N}}{\inf}\left\{\sum_{i=1}^{N}\frac{1}{n_{i}}\sum_{j=1}^{K}\sum_{k=1}^{n_{ij}}\frac{e^{\beta_{i}\left(x_{k}^{i,j}-z_{i}\right)}-1}{\beta_{i}}+\sum_{i=1}^{N}z_{i}\right\}\\ \; & =\sum_{i=1}^{N}\frac{1}{\beta_{i}}\log\left(\frac{1}{n_{i}}\sum_{j=1}^{K}\sum_{k=1}^{n_{ij}}e^{\beta_{i}x_{i}^{j,k}}\right) \end{array}$$

*for any* $\tilde{x}\in R^{n}$ *, where the infimum is arrived at* $z_{i}=\frac{1}{\beta_{i}}\log\left(\frac{1}{n_{i}}\sum_{j=1}^{K}\sum_{k=1}^{n_{ij}}e^{\beta_{i}x_{i}^{j,k}}\right),i=1,\cdots ,N$ *.*

*Note that the penalty functions of multivariate shortfall risk statistic Equation (21) and multivariate divergence risk statistic Equation (23) are equal if we choose* $z_{0}=0$ *. In other words, if* $z_{0}=0$ *, the corresponding loss function and divergence function has a dual relationship.*

**Remark** **4.** $\rho_{\left(g_{1},\cdots ,g_{N}\right)}$ *as in Equation (24) is called multivariate entropic risk statistic, which could be considered as the data-based (or empirical) version of the entropic risk measure in Föllmer and Schied (2002).*

In Example 1, the infimum in Equation (20) and supermum in Equation (22) are hard to calculate by explicit formulas. However, in the next Example 2, we will show that when N=1, the infimum in Equation (20) and supermum in Equation (22) can be expressed by explicit formulas.

**Example** **2.** *For exponential loss function* $\ell \left(x\right):=\frac{e^{\beta x}-1}{\beta },\beta >0$ *, and* $z_{0}>-\frac{1}{\beta }$ *, we know that the conjugation function* $\ell^{*}:\left[0,+\infty \right)$ *is defined as*

$$\begin{array}{c} \ell^{*}\left(x\right)=\left\{\begin{array}{c} \frac{x}{\beta }\log x-\frac{x}{\beta }+\frac{1}{\beta },\;if\;x>0,\\ \frac{1}{\beta },\;\;\;\;\;\;\;x=0. \end{array}\right. \end{array}$$

*Then by the definition of the shortfall risk statistic in Equation (5), we know that the corresponding shortfall risk statistic is*

(25) $$\begin{array}{cc} \rho_{B}\left(\tilde{x}\right) & =\inf\left\{m:\frac{1}{n}\sum_{j=1}^{K}\sum_{k=1}^{n_{j}}\ell \left(x_{jk}-m\right)\leq z_{0}\right\}\\ \; & =\frac{1}{\beta }\left[\log\left(\frac{1}{n}\sum_{j=1}^{K}\sum_{k=1}^{n_{j}}e^{\beta x_{jk}}\right)-\log\left(\beta z_{0}+1\right)\right] \end{array}$$

*for any* $\tilde{x}\in R^{n}$ *.*

*By Theorem 1, we have that the penalty function of* $\rho_{B}$ *is*

$$\begin{array}{cc} \alpha_{min}\left(\tilde{\omega }\right)\; & =\underset{\lambda >0}{\inf}\frac{1}{\lambda }\left\{z_{0}+\frac{1}{n}\sum_{j=1}^{K}\sum_{k=1}^{n_{j}}\ell^{*}\left(\lambda n\omega_{jk}\right)\right\}\\ \; & =\underset{\lambda >0}{\inf}\frac{1}{\lambda }\left\{z_{0}+\frac{1}{n}\sum_{j=1}^{K}\sum_{k=1}^{n_{j}}\left[\frac{\lambda n\omega_{jk}}{\beta }\log\lambda n\omega_{jk}-\frac{\lambda n\omega_{jk}}{\beta }+\frac{1}{\beta }\right]\right\}\\ \; & =\underset{\lambda >0}{\inf}\frac{1}{\lambda }\left\{z_{0}+\frac{1}{\beta }-\frac{\lambda }{\beta }+\frac{1}{n\beta }\sum_{j=1}^{K}\sum_{k=1}^{n_{j}}\lambda n\omega_{jk}\log\lambda n\omega_{jk}\right\}\\ \; & =\frac{1}{\beta z_{0}+1}\left[z_{0}+\frac{1}{\beta }-\frac{\beta z_{0}+1}{\beta }+\frac{1}{n\beta }\sum_{j=1}^{K}\sum_{k=1}^{n_{j}}\left(\beta z_{0}+1\right)n\omega_{jk}\log\left(\beta z_{0}+1\right)n\omega_{jk}\right]\\ \; & =\frac{1}{\beta }\sum_{j=1}^{K}\sum_{k=1}^{n_{j}}\omega_{jk}\log\left[\left(\beta z_{0}+1\right)n\omega_{jk}\right] \end{array}$$

*for any* $\tilde{\omega }\in W=\left\{\left(\omega_{11},\cdots ,\omega_{1n_{1}},\omega_{21},\cdots ,\omega_{Kn_{K}}\right)\in R^{n}:\omega_{jk}\geq 0,\sum_{j=1}^{K}\sum_{k=1}^{n_{j}}\omega_{jk}=1\right\}$ *, where the infimum is arrived at* $\lambda =\beta z_{0}+1$ *.*

*Let* $g\left(x\right):=\ell^{*}\left(x\right)$ *, the divergence function* $I_{g}:{\left[0,+\infty \right)}^{n}\to R$ *is defined by*

$$\begin{array}{cc} I_{g}\left(\tilde{\omega }\right) & =\frac{1}{n}\sum_{j=1}^{K}\sum_{k=1}^{n_{ij}}g\left(n\omega_{jk}\right)\\ \; & =\frac{1}{n}\sum_{j=1}^{K}\sum_{k=1}^{n_{j}}\left(\frac{n\omega_{jk}}{\beta }\log\left(n\omega_{jk}\right)-\frac{n\omega_{jk}}{\beta }+\frac{1}{\beta }\right)\\ \; & =\frac{1}{\beta }\sum_{j=1}^{K}\sum_{k=1}^{n_{j}}\omega_{jk}\log\left(n\omega_{jk}\right). \end{array}$$

*Therefore, by Theorem 3, the divergence risk statistic is*

(26) $$\begin{array}{cc} \rho_{g}\left(\tilde{x}\right) & =\underset{z\in R}{\inf}\left\{\frac{1}{n}\sum_{j=1}^{K}\sum_{k=1}^{n_{j}}g^{*}\left(x_{jk}-z\right)+z\right\}\\ \; & =\underset{z\in R}{\inf}\left\{\frac{1}{n}\sum_{j=1}^{K}\sum_{k=1}^{n_{j}}\frac{e^{\beta (x_{jk}-z)}-1}{\beta }+z\right\}\\ \; & =\frac{1}{\beta }\log\left(\frac{1}{n}\sum_{j=1}^{K}\sum_{k=1}^{n_{j}}e^{\beta x_{jk}}\right) \end{array}$$

*for any* $\tilde{x}\in R^{n}$ *, where the infimum is arrived at* $z=\frac{1}{\beta }\log\left(\frac{1}{n}\sum_{j=1}^{K}\sum_{k=1}^{n_{j}}e^{\beta x_{jk}}\right)$ *.*

*Note that the shortfall risk statistic Equation (25) and divergence risk statistic Equation (26) are equal if we choose* $z_{0}=0$ *. In other words, if* $z_{0}=0$ *, the corresponding loss function and divergence function has a dual relationship.*

**Remark** **5.** *In Example 2, when* $z_{0}=0$ *, then* $\rho_{B}$ *as in Equation (25) is called entropic risk statistic. For general* $z_{0}>-\frac{1}{\beta }$ *,* $\rho_{B}$ *as in Equation (25) is called entropy-like risk statistic.*

## 6. Conclusions

In this paper, we have introduced two new classes of multivariate risk statistics, they are multivariate shortfall and divergence risk statistics. Their basic properties are discussed and presentation results for them are given. Moreover, the coherency of the univariate shortfall risk statistics is characterized. These newly introduced multivariate risk statistics complement the study of risk statistics. Meanwhile, these shortfall and divergence statistics are more tractable than the corresponding risk measures, because they are expressed in terms of data (i.e., samples). It would also be interesting to see the method where the coherency of multivariate shortfall risk statistics is characterized.
